# Free-Energy Landscape of Reverse tRNA Translocation through the Ribosome Analyzed by Electron Microscopy Density Maps and Molecular Dynamics Simulations

**DOI:** 10.1371/journal.pone.0101951

**Published:** 2014-07-07

**Authors:** Hisashi Ishida, Atsushi Matsumoto

**Affiliations:** Quantum Beam Science Directorate and Center for Computational Science and e-Systems, Japan Atomic Energy Agency, Kyoto, Japan; Instituto de Tecnologica Química e Biológica, UNL, Portugal

## Abstract

To understand the mechanism of reverse tRNA translocation in the ribosome, all-atom molecular dynamics simulations of the ribosome-tRNAs-mRNA-EFG complex were performed. The complex at the post-translocational state was directed towards the translocational and pre-translocational states by fitting the complex into cryo-EM density maps. Between a series of the fitting simulations, umbrella sampling simulations were performed to obtain the free-energy landscape. Multistep structural changes, such as a ratchet-like motion and rotation of the head of the small subunit were observed. The free-energy landscape showed that there were two main free-energy barriers: one between the post-translocational and intermediate states, and the other between the pre-translocational and intermediate states. The former corresponded to a clockwise rotation, which was coupled to the movement of P-tRNA over the P/E-gate made of G1338, A1339 and A790 in the small subunit. The latter corresponded to an anticlockwise rotation of the head, which was coupled to the location of the two tRNAs in the hybrid state. This indicates that the coupled motion of the head rotation and tRNA translocation plays an important role in opening and closing of the P/E-gate during the ratchet-like movement in the ribosome. Conformational change of EF-G was interpreted to be the result of the combination of the external motion by L12 around an axis passing near the sarcin-ricin loop, and internal hinge-bending motion. These motions contributed to the movement of domain IV of EF-G to maintain its interaction with A/P-tRNA.

## Introduction

The ribosome is a supra-molecule which synthesizes protein by translating genetic information to the amino acid sequence. During protein synthesis, mRNA and tRNA move through a solvent-accessible channel located at the interface of the large and small ribosomal subunits (50S and 30S, respectively, in bacteria). The large and small subunits have three distinct sites for tRNA to bind at the interface: the A (aminoacyl), P (peptidyl), and E (exit) sites [1] [2]. Protein synthesis is carried out in the elongation cycle of the ribosome, in which there are three main stages: codon-anticodon recognition, peptide-bond formation, and the movement of tRNA and mRNA relative to the ribosome, called translocation [1] [2].

After codon-anticodon recognition, aminoacyl-tRNA (aa-tRNA) is in the classical A/A state, while peptidyl-tRNA is in the classical P/P state. (As for X/Y, where X, Y = A or P or E, and X represents the binding site of the anticodon stem loops (ASL) of tRNAs on the small ribosomal subunit and Y the site of the acceptor ends of tRNAs on the large ribosomal subunit.) Translocation starts after messenger RNA (mRNA) is decoded and peptide bonds are formed by the peptidyl-transferase reaction. During the peptidyl-transferase reaction, the growing polypeptide chain is transferred to the A-site tRNA from the P-site tRNA in classical P/P state, resulting in the formation of the pre-translocational (PRE) complex. In the PRE complex, A-site tRNA and P-site tRNA fluctuate spontaneously between their classical A/A and P/P states and hybrid A/P and P/E states, where the acceptor ends of the tRNAs move with respect to the large subunit while the anticodon ends remain in the A and P sites. [3]. GTPase elongation factor G (EF-G in prokaryotes, eEF2 in eukaryotes) binds to the ribosome in the GTP-bound form and stabilizes the hybrid state [4]. Then, the two tRNAs move to the new classical P/P- and E/E-sites, resulting in the formation of the post-translocational (POST) complex. Translocation finishes with the release of EF-G from the A-site and the release of tRNA from the Esite.

Translocation is thought to be a complex multistep dynamic process which involves both the movement of tRNA through the ribosome and large conformational changes in the ribosome. To understand the dynamics of the ribosome and tRNA during translocation, extensive research using cross-linking analysis [5], pre-steady-state kinetic analysis [6], X-ray crystallography [7] [8] [9] [10] [11], cryo-electron microscopy (cryo-EM) [12] [13] [14] [15] [16] [17] and FRET (fluorescence resonance energy transfer) [18] [19] [20] [21] [22] have been carried out. Cryo-EM experiments indicate that the hybrid formation is accompanied by a ratchet-like movement, more specifically an anticlockwise rotation of the small ribosomal subunit with respect to the large ribosomal subunit as viewed from the solvent face of the small subunit [12]. Single-molecule FRET studies also showed that the formation of the hybrid state was related to the rotated state by the ratchet-like movement [18] [19].

Kinetic analysis showed that GTP hydrolysis on EF-G drives a conformational rearrangement of the ribosome that precedes the movement of tRNA-mRNA [6]. This process is referred to as “unlocking” [6], [13]. After the “unlocking”, GTP hydrolysis and inorganic phosphate (Pi) dissociation lead to translocation of mRNA and tRNA, during which the ribosome reverts to the original non-rotated (POST) state from the rotated state [7]. In the POST state, the ribosome is in the “relocked” state to fix tRNAs predominantly in their new classical P/P- and E/E-sites. It should be noted that there are different uses of the terms “unlocking and relocking”. The different uses are reviewed in detail in a literature of J. Chen [23]. The first unlocking (of the ribosome) happens when peptide bond formation and the ratchet-like movement of the ribosome occur. It should be noted that during this process the position of the ASLs of the tRNAs and mRNA remain locked at the A- and P-sites. The second unlocking (of tRNA-mRNA) occurs when the hydrolysis on EF-G allows the movement of mRNA and the ASLs of the tRNAs. The first relocking (of the ribosome) corresponds to the back ratchet-like movement, while the second relocking (of tRNA-mRNA) locks the movement of tRNA-mRNA to block its reverse movement and to avoid a frameshift during the process. In this study, unlocking and relocking are used in the sense of the second meaning. The state before the unlocking corresponds to the PRE state, while the relocked state corresponds to the POST state. In the PRE state, tRNAs can be either in the classical state (P/P and A/A) or in the hybrid state (P/E and A/P). In this study, we specifically refer to the ribosome bound to EF-G and hybrid tRNA as the ribosome in the PRE state. In the POST state, tRNAs are in a new classical state (P/P and E/E). We specifically refer to the ribosome bound to EF-G and tRNA in the new classical state as the ribosome in the POST state.

It is essential to observe the intermediate (INT) state between the PRE and POST states to understand the process of tRNA translocation. Several structures of the ribosome including tRNA and EF-G which are thought to be in the INT state of translocation have been observed by cryo-EM in the ribosome such as EMD-1365 [12], EMD-1799 [15] and EMD-5775 [17] in prokaryote, and EMD-1343 [13] in eukaryote. EMD-1799, EMD-5775 and EMD-1343 showed a large head motion around the neck region of the small subunit. As the direction of the head movement of the small subunit at the region facing the tRNAs is the same as the direction of tRNA movement from the A to P and P to E site, the head rotation is thought to facilitate the movement of the tRNAs from the hybrid to a new classical state [13] [15] [17]. In contrast to the ordinary translocation facilitated by EF-G, reverse translocation is facilitated by EF-4 (former name LepA) [24] [25]. Under certain conditions (for example, the addition of deacylated tRNAs cognate to the codon at the E-site), it has been shown that tRNA-mRNA can spontaneously move backward from the POST to PRE state in the absence of EF-G [26] [27] or in the presence of EF-G, GTP and antibiotics [28]. The large head-swivel from the POST to PRE state was not observed in the time-resolved EM experiment of reverse translocation [26]. This may indicate that the dynamics of the ribosome during reverse translocation is different from that in ordinary translocation. (Hereafter, ordinary translocation is simply referred to as “transloction” and reverse translocation is referred to as “r-translocation” unless they are specifically mentioned otherwise.)

Recent developments in computational capacity enable us to extract new information about the dynamics of the ribosome from the increasing amount of experimental data available [29] [30] [31] [32] [33] [34]. However, little is known at the atomic level about how the conformational changes of the ribosome occur in r-translocation. Our motivation in this study is to obtain the free-energy landscape for r-translocation by observing the conformational changes of the ribosome-tRNAs-mRNA-EFG complex from a structure in the POST state determined by X-ray crystallography to structures in the INT and PRE states determined by cryo-EM. In this study, we employed an EM-fitting method using molecular dynamics (MD) simulation [35] [36] [37] to trace the pathway of r-translocation. Along the pathway induced by EM-fitting, umbrella sampling simulations were performed to obtain the free-energy landscape of r-translocation. The relationship between the free-energy and the conformational transition of the ribosome were analyzed. The conformational transition of EF-G was also analyzed. Finally, a model of r-translocation based on the X-ray structure and EM maps is proposed.

## Materials and Methods

### Preparation for the initial atomic structure and EM density maps

To prepare the initial atomic structure for the fitting, we used the atomic structure of *Thermus thermophilus* 70S ribosome in the POST state (containing the large subunit 50S, the small subunit 30S, two tRNA molecules, an mRNA and EF-G) which was determined by X-ray crystallography (PDB code: 2WRI/2WRJ (the former and latter are the small and large subunits, respectively), resolution: 3.6 Å) [10]. The unseen parts of L12 of 70S ribosome in 2WRJ were modeled by using another structure of *Thermus thermophilus* 70S ribosome (PDB code: 1YL3, resolution: 5.5 Å) [38]. The unseen parts of EF-G were modeled by using a homologous molecule from *Thermus thermophilus* EFG2 (EFG-like protein, PDB code:1WDT, resolution: 2.2 Å) [14]. 5-methyluridines in tRNA molecules were changed to uridine. Magnesium and zinc ions and a GDP molecule binding to EF-G remained but fusidic acid was removed. The codon of AAA at the E-site, which does not interact with the anticodon of the E-tRNA, UAC in the X-ray structure, was changed to AUG to be able to bind to UAC in the INT and PRE states. Hydrogen atoms were added in order to construct an all-atom structure of the ribosome-tRNAs-mRNA-EFG complex. This system was composed of 267,362 atoms.

We used the target EM data of *Escherichia coli* 70S ribosome complexed with the P/E-tRNA, an mRNA and EF-G; EMD-1365 (resolution: 11.75 Å, voxel size: 2.82 Å) and EMD-1363 (resolution: 10.9 Å, voxel size: 2.82 Å) observed in the INT and PRE states, respectively. The data were obtained from the electron microscopy database at the European Bioinformatics Institute; EMD-1365 observed in the presence of puromycin, GDP and fusidic acid (resolution: 11.75 Å, voxel size: 2.82 Å), and EMD-1363 observed in the presence of puromycin and GDPNP (resolution: 10.9 Å, voxel size: 2.82 Å) corresponding to the INT and PRE states, respectively [12]. Since the structure of *Thermus thermophilus* 70S ribosome is generally considered to be similar to that of *Escherichia coli* 70S ribosome, the EM density maps of *Escherichia coli* 70S ribosome were used.

### Energy minimization of the initial atomic structure in vaccum

The energy minimization was carried out using an MD simulation program called SCUBA [30] [36] [39] [40] with the AMBER parm99SB force-field [41]. To carry out energy minimization of the system to alleviate unfavorable interactions in the system, steepest descent was performed for 1000 steps, and conjugate gradient was performed for 5000 steps. Harmonic restraints with a force constraint of 1.0 kcal/mol/Å^2^ were applied to all the heavy atoms of the molecules. The distance-dependent dielectric constant of 4.0*r* with the value of *r* in Angstrom was used, and the van der Waals interactions were evaluated with a cut-off radius of 14 Å.

### Molecular dynamics simulation with explicit water molecules

All the MD simulations were performed using an MD simulation program called SCUBA [30] [36] [39] [40] with the AMBER parm99SB force-field [41]. Explicit water was used to observe the conformational change of the ribosome in a realistic way. After the rigid-body fitting simulation, the system of the ribosome-tRNAs-mRNA-EFG complex was placed in a rectangular box 280 Å×320 Å×270 Å. In this box, all the atoms of the complex were separated more than 15 Å from the edge of the box. To neutralize the charges of the system, magnesium ions were placed at positions with large negative electrostatic potential. Moreover, excess ions, including magnesium, potassium, and chloride, were added in the box at concentrations of 100 mM KCl and 7 mM MgCl_2_. After the neutralization of the system, 611,497 TIP3P water molecules were added to the system. Subsequently, the system of the ribosome-tRNAs-mRNA-EFG complex comprised 2,107,110 atoms including GDP, 4 Zn^2+^, 2142 Mg^2+^, 1436 K^+^ and 1635 Cl^−^ ions. The dielectric constant used was 1.0 and the van der Waals interactions were evaluated with a shifted-force cut-off at 9 Å. The nonbonded list, including neighboring atoms within 9 Å, was updated every 50 fs. The particle-particle particle-mesh (PPPM) method [42], [43] was used for the electrostatic interactions with a direct space cutoff of 9 Å. For the PPPM calculations, charge grid sizes of 256×320×256 were chosen for the ribosome system to set the charge grid spacing close to 1 Å. The charge grid was interpolated using a spline of the order of seven, while the force was evaluated using a differential operator of the order of six [43].

The energy minimization of the system in the aqueous medium was carried out using a harmonic restraint with force constraint of 1.0 kcal/mol/Å^2^ for all the heavy atoms of the molecules in the ribosome-tRNAs-mRNA-EFG and bound ions. Then the system was heated from 0 K to 300 K within 500 ps, during which the water molecules and excess ions were allowed to move freely. The system was equilibrated for 3 ns with decreasing restraints on the molecules for 1 ns, and for 2 ns with no restraint at a constant pressure of one bar and a temperature of 300 K. Then the box-size was fixed, and the system was equilibrated for a further 2 ns at a constant temperature of 300 K. During the equilibration the systematic artificial movements of the translational and rotational movements of the ribosome-tRNAs-mRNA-EFG complex which came from the simulation settings were removed. The constant temperature and pressure algorithm developed by Martyna et al. [44] was used to control the temperature and pressure of the system. During the equilibration in the constant pressure and temperature simulation, the weights of barostat and thermostat were set at 10^7^ ps^2^ kcal/mol so that the relaxation times for the temperature and pressure control were both ∼10 ps. After the box-size was fixed, the weight of barostat was set at 10^9^ ps^2^ kcal/mol so that the relaxation time for the temperature was ∼ ps. To integrate the equation of motion, the multi-time-step (MTS) algorithm [45] developed by Zhou et al. was used with a time step of 1 and 2 fs for short and medium forces and 4 fs for other long-range forces. Forces associated with bonded interactions and nonbonded interactions within the cutoff length were classified as short and medium forces, respectively. Forces associated with the electrostatic interactions outside the cutoff length calculated as reciprocal-space interactions by the PPPM method were classified as medium and long-range forces as described in the literature of Zhou et al. [45].

### Algorithm of the EM-fitting simulation

In order to fit the atomic model into the EM density map using MD simulation, we set a target function which is used to constrain the whole structure into an EM density map, in addition to the standard all-atom energy function which imposes physical constraints on the structure.
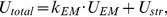
(1)where, *U_str_* is the standard all-atom energy function, and *k_EM_* is a constant weight value to determine how much EM-fitting force should be included in the simulation. *U_EM_* is the function to fit the modeled structure into the EM density map. Here we implemented the EM-fitting method developed by Orzechowski and Tama [37] in SCUBA. In their method, *U_EM_* is the real-space correlation coefficient (CC) of the simulated and computed densities times −1 [37];
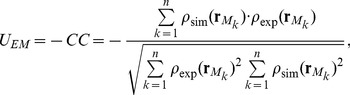
(2)where 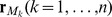 is the coordinate at a *k*-th voxel point. *n* is the total number of voxel points in the system. 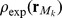 are the experimental EM densities at 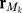. In this study, all the density values less than zero were set at zero. 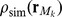 are the simulated EM densities at 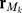 calculated from a given set of atomic coordinates. This is represented as:
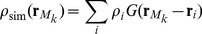
(3)where *ρ_i_* is the atomic mass in the atomic mass unit and 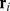 is the atomic coordinate of atom *i*. *G* is the Gaussian function which distributes *ρ_i_* to the voxel points around the atom. 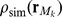 is calculated as the sum of the contributions from the neighboring atoms. The resolution of the EM density map we used here is defined by half the width of a Gaussian in Fourier space. In this study, the atomic mass was distributed by the Gaussian function to the three or four nearest grids (*N*_neiboor_ = 3 or 4) in the negative and positive directions in the three dimensional space. Using *N*_neiboor_ = 4 gives a result which has 4 significant digits of the CC. The force derived from the derivative of the CC is described as:
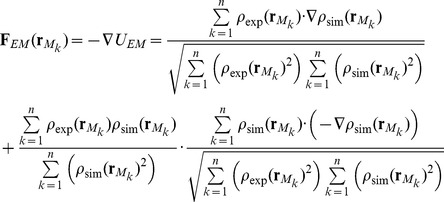
(4)


It should be noted that the force in Eq. (4) is not calculated at the atomic position but at the voxel points. The force at each particle is given as:
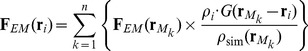
(5)In this study, The total force which should be imposed on the atomic positions can be described as:
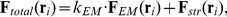
(6)where 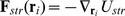 is the atomic interaction force. We call 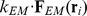 an EM density force. To adjust the strength of the EM density force in the EM-fitting simulation, it is convenient to use *w_EM_* :
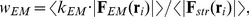
(7)where < > shows the average among atoms. If *w_EM_* = 1. 0 then the averaged magnitude of the EM-fitting force used in EM-fitting simulations, is the same as that of **F** *_str_*.

### Alignment of the EM density maps to the initial atomic model

As the original EM structures of EMD-1365 and EMD-1363 are located differently with regards to the initial atomic model, the target EMD-1365 and EMD-1363 were aligned to the initial atomic model as much as possible. First, we used a rigid-body fitting method to fit the initial atomic structure into the EM density maps. The voxel points with positive values in the EM density maps were selected so that the volume occupied by the voxel points was equivalent to the molecular volume. The model was fitted into the map by matching moments of inertia between the atomic model and the selected voxel points. Moreover, the structure was slightly translated and rotated until the position and orientation of the structure fitted into the target EM density maps as closely as possible [31]. Second, each atomic model fitted into EMD-1365 and EMD-1363 was re-fitted into the EM density map again as a rigid-body using an MD simulation, in which the target function of the CC of Eq. (2) was used. Third, the orientation and translation between the initial atomic structure and the structure re-fitted in each EM density map was measured. Fourth, the same orientation and translation were applied to each EM density map to align it to the initial atomic structure. Finally, as the voxel points of these EM density maps were not located on the orthogonal coordinate system anymore, the data on the voxel points were distributed to the neighboring grid points on the orthogonal coordinate system using the CIC (cloud in cell) method [42].

### The protocol of EM-fitting and umbrella sampling simulations

**Figure 1:**
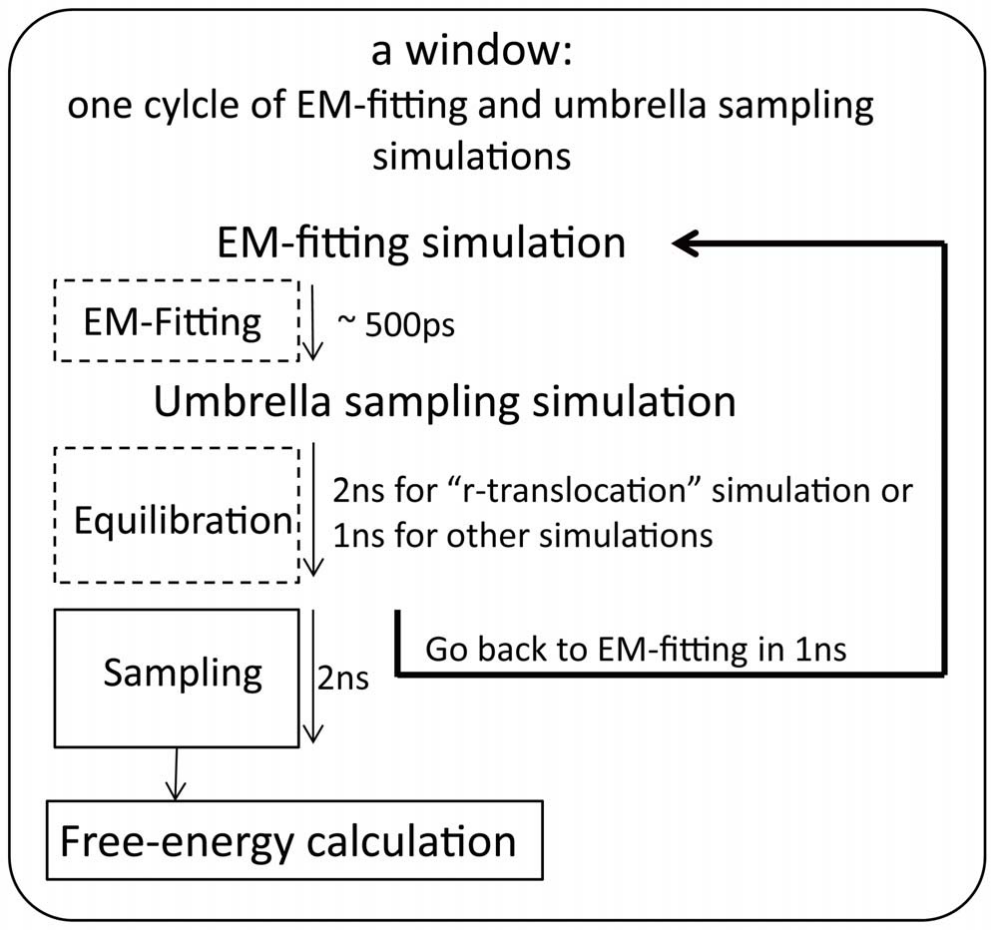
The protocol of the EM-fitting and the umbrella sampling simulations. Schematic representation of the protocol of the EM-fitting and the umbrella sampling simulations to obtain the free-energy landscape is shown.

Fig. 1 shows a schematic representation of the protocol of EM-fitting and umbrella sampling simulations. The EM-fitting simulation was used to trace the translocation pathway by deforming the conformation from the POST to PRE states. *w_EM_* in Eq. (7) should be large enough to move the atomic model into an EM density map, while at the same time it should also be small enough not to overfit the atomic model too much. In this study, the weight of the EM-fitting force started with *w_EM_* = 1.0×10^−7^ and finished with less than 1.0×10^−4^ to maintain the secondary structures during the EM-fitting. As the EM-fitting force did not change very quickly in several fs, the time step for the EM-fitting force was set at 8 fs for *N*_neiboor_ = 3, and 32 fs for *N*_neiboor_ = 4 in the the MTS algorithm.

The umbrella sampling simulation was performed after the EM-fitting simulation. Biased harmonic potentials were imposed on the centers of mass of 50 constitute molecules in the ribosome-tRNAs-mRNA-EFG complex (5S rRNA in the small subunit, all ribosomal proteins excluding L10 and L12, and EF-G) to maintain the slightly deformed structure, generated by the EM-fitting simulation, in order to sample their trajectories for the free-energy calculation. The biased potentials were not imposed on 16S rRNA in the small subunit and 23S rRNA in the large subunit to allow them to move freely and sample their coordinates as much as possible. (Hereafter, these centers of mass are referred to as “umbrella coordinates”.) Then the EM-fitting and umbrella sampling simulations were repeated again. One cycle of these simulations is called a window.

In each window, the EM-fitting simulation was performed for ∼500 ps on average. The EM-fitting forces were applied on all the atoms of the complex in the “classical tRNA” simulation (see Text S1 “The free-energy landscape in the classical state of tRNA (“classical tRNA” simulation)”), while the EM-fitting forces on tRNAs were set to zero in other simulations including the “r-translocation” simulation. The following umbrella sampling simulation, first to equilibrate the system for 1 or 2 ns and then to sample the umbrella coordinates for 2 ns, was performed. All the force constants of the biased harmonic potentials were set to be 1.0 kcal/mol/Å^2^. Each biased harmonic potential gives each center of mass imposed by the potential a root mean square of fluctuation (RMSF) of ∼0.2 Å. During the sampling phase, the umbrella coordinates and the conformation of the ribosome-tRNAs-mRNA-EFG complex were stored every 0.1 ps and 1 ps, respectively. The EM-fitting simulation in the next window was started using the configuration of the system at 1 ns in the sampling phase in the previous window. This procedure was repeated until the structure was close enough to the respective state of EMD-1365 and EMD-1363 (see Fig. S1). To make the sampled trajectories in successive umbrella sampling simulations overlap each other, *w_EM_* was adjusted during the EM-fitting simulation to restrict the displacement of each umbrella coordinate in the previous and present EM-fitting simulations to be within 0.60 Å, which was small enough to overlap the sampled umbrella variables with those in the previous and following umbrella sampling simulations. The umbrella coordinates whose magnitudes were more than 15.0 Å in the iterative simulations of ∼60 windows were those of protein S7 (17.4 Å), S9 (15.4 Å), S13 (19.7 Å), S19 (19.1 Å) and THX (15.3 Å). Details concerning the protocol of the EM-fitting and umbrella sampling simulations are given in Table 1.

**Table 1:** The summary of the protocol of the EM-fitting and umbrella sampling simulations.

Simulation	Target EMD	Number of windows	Simulation time for each window [ns]	Simulation time [ns]	Number of windows used for free-energy calculation	Correspondences with W (the window number in Fig. S2), and the definition of R^f^	Inner product between R^f^ and R^f^ in the “reverse translocation”
1. Initial equilibration				5.5			
2. “classical tRNA” in Fig. S2(a)	1365	42	0.5^a^+1.0^b^+2.0^c^ = 3.5	147.0	42	W = 1–42, **R** ^f^ = <**R**>_W = 42_ (R_1_(**R** ^f^) = 30.8 Å)	0.910
3. “classical tRNA” in Fig. S2(b)	1363	18	3.5	63.0	14^A^+18 = 32	W = 15–32, **R** ^f^ = <**R**>_W = 32_ (<**R**>_W = 14_ = 14.0 Å)	0.904
4. “classical tRNA” in Fig. S2(c)	1363	12	3.5	42.0	20^B^+12^C^ = 32	W = 21–32, **R** ^f^ = <**R**>_W = 32_ (<**R**>_W = 20_ = 18.3 Å)	0.938
5. “classical tRNA” in Fig. S2(d)	1363	7	3.5	24.5	25^D^+7 = 32	W = 26–32, **R** ^f^ = <**R**>_W = 32_ (<**R**>_W = 25_ = 21.9 Å)	0.906
6. “classical tRNA” in Fig. S2(e) (or Fig. S4(a))	1363	25	3.5	87.5	20^B^+12^C^+25 = 57	W = 33–57, **R** ^f^ = <**R**>_W = 46_ (R_1_(**R** ^f^) = 36.1 Å)	0.957
7. Modeling “semi-hybrid tRNA”				10.0			
8. “semi-hybrid tRNA” in Fig. S4(b)	1365 and 1363	6 and 23	3.5	101.5	29	(Figure not shown)	
9. Modeling “hybrid tRNA”				10.0			
10. “hybrid tRNA” in Fig. S4(c)	1363	16	3.5	56.0	16	(Figure not shown)	
11. “r-translocation” in Fig. 2 (or Fig. S2(f))	1365 and 1363	10 and 38	0.5^a^+2.0^b^+2.0^c^ = 4.5	216.0	14^A^+10+38 = 62	W = 15–24, 25–62, **R** ^f^ = <**R**>_W = 52_ (<**R**>_W = 24_ = 18.5 Å, R_1_(**R** ^f^) = 36.9 Å)	1.000
12. “r-translocation” (second run) in Fig. 2 (or Fig. S2(g))	1365 and 1363	10 and 33	3.5	150.5	14^A^+10+33 = 57	W = 15–24, 25–57, **R** ^f^ = <**R**>_W = 50_ (<**R**>_W = 24_ = 19.8 Å, R_1_(**R** ^f^) = 36.5 Å)	0.969
13. effect of different ion force-fields in Fig. S2(h)		33	3.5	115.5	14^A^+10^E^+33 = 57		
14. effect of different umbrella variables in Fig. S7				32.0			
Total				1061.0			

a, b and c in “Simulation time for each window” are the simulation times for the EM-fitting, equilibration and umbrella sampling simulations, respectively. A, B, C and D in “Number of windows used for free-energy calculation”, which were out of 42 windows obtained at “2. “classical tRNA” in Fig. S2 (a)”, were reused for each free-energy calculation. E in “Number of windows used for free-energy calculation”, which was obtained at “12. “r-translocation” (second run) in Fig. 2 (or Fig. S2(g))”, were reused for the free-energy calculation using a corrected ion force-field. **R** ^f^ was defined as the average position of **R**, <**R**>_W = *N*_ in the *N*-th window.

### Free-energy profile using multi-dimensional WHAM

The weighted histogram analysis method (WHAM) [46] was used to obtain the free-energy landscape from the sampled trajectories in the umbrella sampling simulations. In the WHAM approach, the unbiased probability distribution *P*(**R**) is calculated from the biased probability distribution of the sampled umbrella coordinates as:
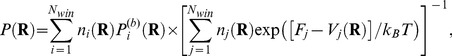
(8)where **R** is the *n_biased_*×3 dimensional vector which comprises the umbrella coordinates, 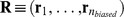, *N_win_* is the number of windows, *n_i_*(**R**) is the number of data points in the *i*-th window, *P_i_^(b)^*(**R**) is a biased probability from the raw data obtained in the umbrella sampling simulation, *V_j_*(**R**) is the sum of the *n_biased_* ( = 50) biasing potentials in the *j*-th window, *k_B_* is the Boltzmann constant, and *T* is the temperature. The coefficient *F*_j_ is defined by:
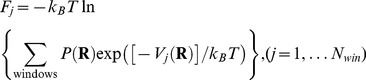
(9)where the summation includes all the coordinates of **R** which were sampled over the total number of windows. By iterating Eqs. (8) and (9) to achieve self consistency (using a tolerance of 10^−4^), the relative free-energy *F*(**R**) at a given **R** is obtained as:
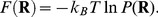
(10)


Although the free-energy can be calculated from Eq. (10), it is impossible to visualize the calculated free-energy profile in the full *n_biased_*×3 dimensions. In this study the *n_biased_*×3 dimensions were reduced to 1 or 2 dimensions by defining an appropriate coordinate (reaction coordinate) on which all the umbrella coordinates were projected. The first reaction coordinate of translocation, *R_1_*, was defined as follows:
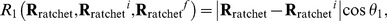
(11)where
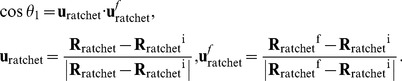
(12)
**R**_ratchet_ is a subset of **R** related to the ratchet-like movement selected from the umbrella coordinates. In this study, the coordinates of **R**_ratchet_ are chosen from all the umbrella coordinates except for six proteins (L10, four L12s and EF-G). **u**_ratchet_ and **u**_ratchet_^f^ are the (*n_biased_*-6)×3 dimensional unit vector of **R**_ratchet_ - **R**_ratchet_^i^, and **R**_ratchet_^f^ - **R**_ratchet_^i^, respectively. **R**_ratchet_^i^ and **R**_ratchet_^f^ are the initial and the targeted final positions of the umbrella coordinates of **R**_ratchet_. The X-ray structure of 2WRI/2WRJ as the initial structure is at *R_1_* = 0 Å. *R_1_* is the 1-dimensional coordinate obtained by projecting the vector (**R**_ratchet_ - **R**_ratchet_^i^) in the direction of (**R**_ratchet_^f^ - **R**_ratchet_^i^). This reaction coordinate is thought to be a good indicator of how far ratchet-like movement has progressed from the initial state. When *R_1_* is calculated, the position of **R**_ratchet_, which was superposed on that of **R**_ratchet_^i^, was used.

To understand how P-tRNA, E-tRNA and EF-G move thorough the ribosome with regards to the reaction coordinate of *R_1_*, the centers of mass of the anticodons of P-tRNA and E-tRNA, and the center of mass of domain IV (residues 481–603 and the C-terminal region 675–690) of EF-G were used as the second reaction coordinate, *R_2_*. The second reaction coordinate of translocation, *R_2_*, was defined as follows:
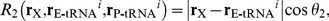
(13)where
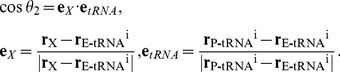
(14)
**r**_X_ is the positon of the center of mass of X, where X is the P-tRNA, the E-tRNA and EF-G to represent them respectively. **r** *_E-tRNA_^i^* and **r** *_P-tRNA_^i^* are the initial positions of E-tRNA and P-tRNA in the initial structure, respectively. **e**_x_ and **e**_tRNA_ are the 3-dimensional unit vectors of **r**_X_ - **r** *_E-tRNA_^i^*, and **r** *_E-tRNA_^i^* - **r** *_P-tRNA_^i^*, respectively. The sign of *R_2_* was defined as positive when it moves in the direction of translocation from the P- to E-site.

The probability of the trajectories on *X*, *P*(*X*), can be written as:
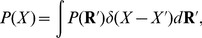
(15)where *δ(X)* is the Dirac delta-function, and the free-energy profile in 1-dimension has the same form as Eq. (10):
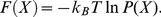
(16)


To describe the changes in a physical quantity, *A*, such as the distance between atoms along *X*, the averaged quantity at *X*, 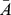
(*X*), is calculated by weighing the unbiased probability on the quantity *A*(*R*) as:
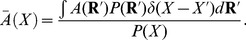
(17)The root mean square deviation (rmsd) from 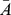
(*X*) is
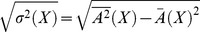
(18)The probability and the 2-dimensional free-energy landscape with regards to *X* and 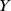,* P*(*X*,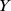) and *F*(*X*,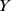) respectively, are expressed as:
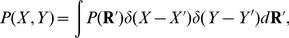
(19)

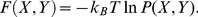
(20)


### The free-energy calculations in the “classical tRNA”, “semi-hybrid tRNA” and “hybrid tRNA” states

Fig. S2 shows the free-energy of the “classical tRNA” simulation. However, as tRNA translocation did not occur in the “classical tRNA” simulation” (Fig. S3), the translocational path was predicted to be a path connecting local free-energy minimum obtained from the “classical tRNA”, “semi-hybrid tRNA” and ‘hybrid tRNA” simulations (Fig. S4). (The schematic representation of the “classical tRNA”, “semi-hybrid tRNA” and “hybrid tRNA” states in respective simulations is shown in Fig. S5) Details of the protocol for these simulations and the location of these free-energy minima are given in Texts S1 “The free-energy landscape in the classical state of tRNA (“classical tRNA” simulation)”, S2 “Modeling of the semi-hybrid and hybrid states of tRNAs” and S3 “The free-energy landscape in the semi-hybrid and hybrid states of tRNA (“semi-hybrid tRNA” and “hybrid tRNA” simulations)”.

### Path-search algorithm for the “r-translocation” simulation

A path-search algorithm [39] was utilized to search for a minimum free-energy path (MFEP) which connects the free-energy minima in “classical-tRNA”, “semi-hybrid tRNA” and “hybrid tRNA” states. MFEPs are often quantities of most interest because the MFEPs are often identified with the most likely reaction paths. It is known that transitions are most likely to occur around the MFEP; given a set of collective variables to describe a reaction (in this study the umbrella sampling variables), the MFEP is the reaction path of maximum likelihood in these variables [47].

Using the path-search algorithm, two harmonic potentials were imposed to induce the movement of the two tRNAs from the E- and P-sites to the P- and A-sites on the small subunit during the EM-fitting simulation in the “r-translocation” simulation. In studies of FRET [22], cryo-EM [26], normal mode analysis [29] and molecular dynamics simulation [34], it is suggested that the translocation of tRNA (or mRNA) and the ratchet-like movement of the ribosome are coupled. Therefore, the induction of these movements at the same time would be appropriate to find MFEP for r-translocation.

To induce an appropriate movement of the tRNAs, the force required to activate the movement of tRNAs was assumed to always work in the direction of the targeted position of the lowest free-energy minima in the “semi-hybrid tRNA” or “hybrid tRNA” states. To apply this assumption to a series of EM-fitting simulations, two harmonic potentials were imposed on the anticodons of the two tRNAs, along a line between the present and targeted position at the INT or PRE state. The center of the potential for **r** *_i,n_* (*i*-th coordinate of the anticodons of tRNAs of the P- and E-sites (*i* = 1, 2) in the *n*-th EM-fitting simulation) was set at the desired position, 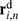, as follows:
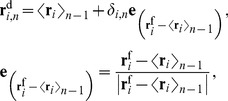
(21)where <***r*** *_i_*>_n-1_ is the average position of the *i*-th coordinate sampled during the first 1 ns in the sampling phase in the (*n*-1)-th window. **r** *_i_* ^f^ is the targeted position in the semi-hybrid state before the system reaches the semi-hybrid state, while **r** *_i_* ^f^ is the targeted position in the hybrid state after the system reached the semi-hybrid state. 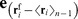 is the 3-dimentional unit vector from <***r*** *_i_*>_n-1_ in the direction of **r** *_i_* ^f^. δ*_i,n_* is an arbitrary distance parameter which is set to move **r** *_i,n_*, with a length of δ*_i,n_*, from <***r*** *_i_*>_n-1_ in the direction of **r** *_i_* ^f^. δ*_i,n_* should be set small enough so that the potential can move **r** *_i,n_* slowly towards **r** *_i_* ^f^ without causing severe steric strain. The harmonic potential, *V_i,n_*, to induce the movement of **r** *_i,n_* to 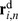, has the form:
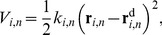
(22)where *k_i,n_* is an arbitrary harmonic force constant. *k_i,n_* should be set large enough so that tRNAs can translocate, but small enough so that it does not cause unnatural movement. To give *k_i,n_* an appropriate value, the projection of <***r*** *_i_*>_n_ - <***r*** *_i_*>_n-1_ onto 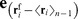 was measured at 1 ns of the sampling phase in the *n*-th window, as follows:
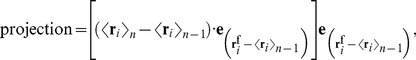
(23)This projection indicates the magnitude of the movement of the *i*-th coordinate from <***r*** *_i_*>_n-1_ to <***r*** *_i_*>_n_ in the direction of 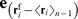. Here, another arbitrary distance parameter, Δ*_i,n_*, is introduced. If the projection is less than Δ*_i,n_*, then *k_i,n_*_+1_ for the EM-fitting simulation in the (*n*+1)-th window is increased to facilitate the movement of tRNAs; however, if the projection is larger than Δ*_i,n_*, then *k_i,n_*_+1_ is decreased so as not to apply an unnecessarily strong potential in the EM-fitting simulation in the (*n*+1)-th window. Thus, each of the two force constants used to facilitate tRNA translocation is adjusted according to the environment around the tRNAs. In this study, the parameter δ*_i,n_* was adjusted within the range of 0.3 and 0.6 Å in each EM-fitting simulation by monitoring the smooth movement of the tRNAs. The parameter Δ*_i,n_* was set at δ*_i,n_*/2. The force constant of the potential, *k_i,n_*, was increased or decreased by 25 pN/Å (1 pN = 1.439×10^−2^ kcal/mol/Å) for the tRNAs, according to whether the projection in Eq. (23) was larger than Δ*_i.n_* or not. The initial value of the force constant of the potential, *k_i,n_*_ = 1_, was set at 100 pN/Å. The potential was gradually removed in the first 1 ns during the following equilibration simulation for 2 ns.

In addition to the harmonic potentials for the path-search algorithm, the two different kinds of restraint were applied: (1) RMSD restraints for each tRNA and domain IV of EFG within 1 Å from their last structures in the previous umbrella sampling simulation, (2) flat-bottomed artificial distance restraints for hydrogen bonds between the anticodons of the two tRNAs and the corresponding codons of mRNA. Only when the hydrogen bond distance deviated more than 0.1 Å from its ideal value, did the flat-bottomed artificial distance restraint start to work to bring it back. In the next equilibration simulation, these harmonic potentials and restraints were removed in the first 1 ns of the equilibrium simulation which lasted for 2 ns. This was followed by a 2-ns simulation to sample the umbrella variables to calculate the free-energy.

The “r-translocation” simulation was carried out twice to assess the difference in the free-energy landscapes along different translocational paths in the INT state by changing the start time of the path-search algorithm at *R_1_* = ∼15 and 16 Å in the first and second runs respectively, and changing the simulation time of the EM-fitting simulation. The EM density data used was switched from EMD-1365 to EMD-1363 in the EM-fitting simulation at the 25-th window (where the average position of *R_1_* in the previous 24-th window, <*R_1_*>_W = 24_, was 18.5 and 19.8 Å in the first and second runs, respectively (see Table 1 and Fig. S1(d)).

### The definitions of angles of the ratchet-like movement of the ribosome and the rotational movement of the head

The rachet-like angle in this study was defined as follows; the unit vector of the ratchet-like movement, **e**_ratchet_, was set from the center of mass of the small subunit (16S rRNA and proteins contributing to **R**_ratchet_) to that of the large subunit (23S and 5S rRNAs and proteins contributing to **R**_ratchet_). The atomic structures of the ribosome were best-fit in the initial (energy-minimized) structure by fitting on the large subunit (structure A). The rotational vector 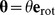 which best-fits the small subunit of structure A in that of the initial structure along the unit vector of **e**_rot_ by the angle of *θ* was calculated. The direction of **e**_rot_ was set to be most parallel to be **e**_ratchet_. The magnitude of the ratchet-angle *θ*_ratchet_ was calculated as 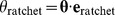


The angle of rotation of the head was defined as follows: the unit vector of the head, **e**_head_, was defined as the unit vector of the third principal axis of the head region (G917-A1396 in 16S rRNA, proteins S3, S7, S9, S13, S14, S19, THX) of the small subunit, which was most parallel to the unit vector of tRNA translocation, **e** *_tRNA_*, in Eq. (14) among the three principal axes. The direction of the angle of the head is chosen to be positive when the head rotates clockwise. The magnitude of rotation of the head was calculated as the angle between **e** *_tRNA_* and the projected line of **e**_head_ on the plane made of **e** *_tRNA_* and **e**_ratchet_.

### Conformational analysis of EF-G

DynDom [48] [49] is able to determine dynamic domains, hinge axes, and hinge-bending residues from two biomolecule structures that have different conformations. The internal motion was defined as the displacement between them after best-fitting the structure in the POST to that in the PRE state, and the external motion was defined as the residual displacement. To analyze the inter-domain motions in the internal motion of EF-G, DynDom utilizes Chasles' theorem in kinematics [50] which states that “the most general rigid body displacement can be produced by a translocation along a line (called its screw axis) followed (or preceded) by a rotation about that line”. The description of the displacement involves the determination of parameters, which specify the straight line coincident with the screw axis, the angle of rotation about the axis and the translation along the axis [48] [49]. The rotational axis and the magnitude of the translation along the axis in the external motion of the whole EF-G through the ribosome were also analyzed using Chasles' theorem. The formula to calculate the rotational axis and the magnitude of the translation along the axis is described in the literature of Hayward [48] [49] and Chasles [50] in detail.

### Computational time

All the simulations covering a total time of more than 1 µs were performed (Table 1) on the Fujitsu BX900 and FX1 supercomputers at Japan Atomic Energy Agency. The total computational time was more than 10 million CPU core hours.

## Results

### Free-energy landscape of r-translocation

Fig. 2 shows the free-energy of “r-translocation”. (Hereafter, for the sake of convenience, we call the state at *R_1_* = ∼0–18 Å, ∼19–33 Å and ∼34–44 Å the POST, INT and PRE states. Fig. S6 shows the linear relationship between *R_1_* and the ratchet angle, indicating that *R_1_* is a good indicator of the ratchet-like movement of the ribosome.) The POST and PRE states had a global and local minimum at *R_1_* = 15 Å and ∼34–36 Å, respectively. There were two main energy barriers of ∼7–9 kcal/mol between the POST and INT states (*R_1_* = ∼19–22 Å), and ∼8 kcal/mol between the INT and PRE states (*R_1_* = ∼30–33 Å).

**Figure 2:**
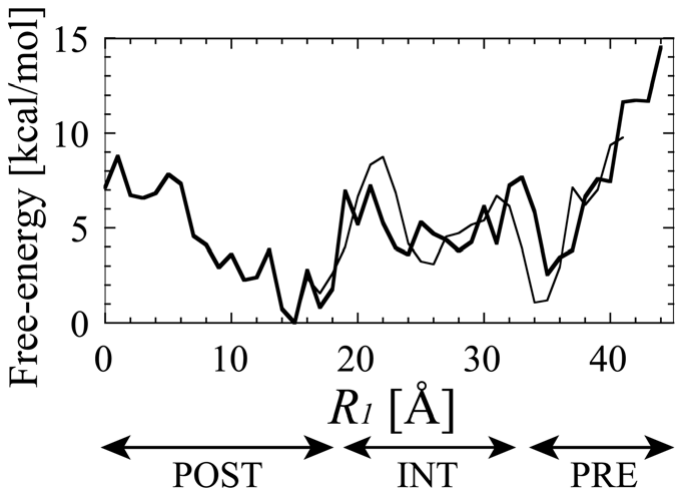
The free-energy landscape of reverse tRNA translocation. The free-energy landscapes obtained from the first and second “r-translocation” simulations are plotted against *R_1_* with thick and thin lines, respectively. The lowest free-energy was set at zero for each case. The average of the absolute difference between each value of their free-energies in the range of *R_1_* = 17–41 Å was 2.2 kcal/mol. For the sake of convenience, the POST, INT and PRE states are leveled at *R_1_* = ∼0–18 Å, ∼19–33 Å and ∼34–44 Å, respectively. The units of the reaction coordinate and the free-energy are Å and kcal/mol, respectively.

**Figure 3:**
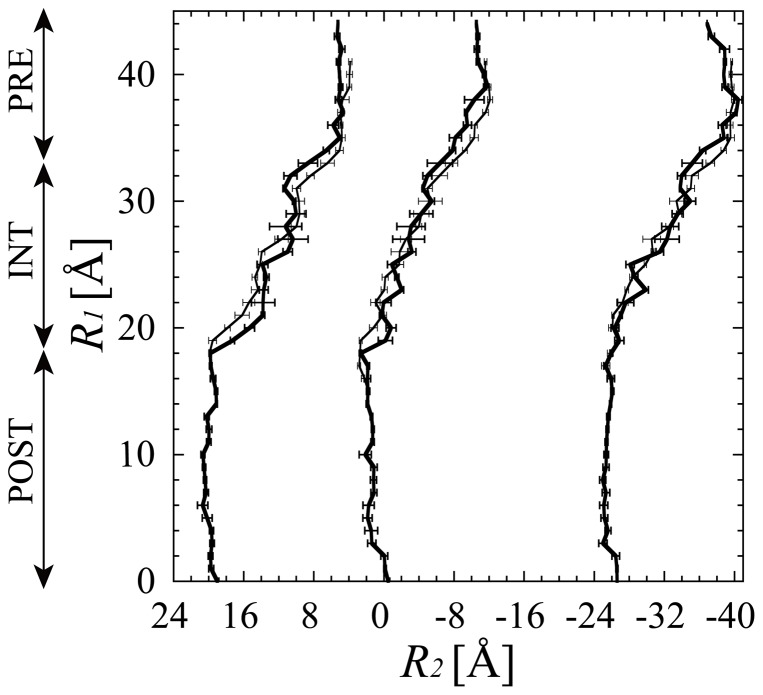
The movements of the P-tRNA, E-tRNA and EF-G. (a) The paths of the centers of mass of the anticodons of the P-tRNA and E-tRNA, and of domain IV (residues 481–603 and the C-terminal region 675–690) of EF-G, the second reaction coordinates of *R_2_*, are plotted against *R_1_*. The paths obtained from the first and second “r-translocation” simulations are plotted with thick and thin lines, respectively.

Fig. S3 shows that the movement of the tRNAs with respect to the small subunit from the classical P/P and E/E states into the hybrid A/P and P/E states was properly directed (see also Text S4 “The verification of the “r-translocation” simulation). Hereafter, the tRNA molecules of the P/P-tRNA and E/E-tRNA in the POST are referred to as “P-tRNA” and “E-tRNA”. The A/P-tRNA and P/E-tRNA in the PRE state are also referred to as “P-tRNA” and “E-tRNA” unless specifically mentioned otherwise. Fig. 3 shows the paths of the P-tRNA and E-tRNA, and of domain IV of EF-G during r-translocation. Their paths show that they moved together in the negative direction of *R_2_* by ∼15 Å, while maintaining the interactions among them from the POST at *R_1_* = 15 Å to PRE state at ∼35–37 Å. (The movement of ∼15 Å is large enough for the P-tRNA and E-tRNA to reach the hybrid state from the classical state, as shown in Fig. S3.)

### The rotation of the head of the small subunit

**Figure 4:**
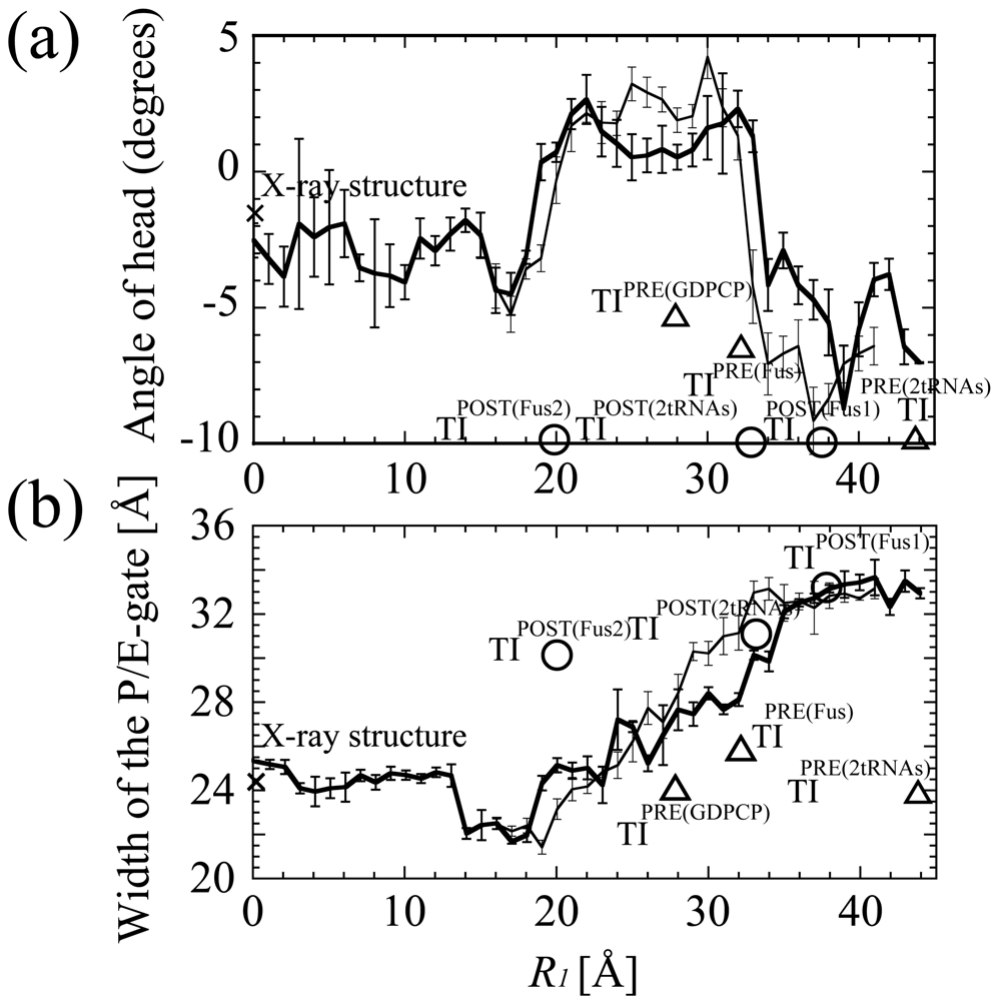
The movements of the head of the small subunit and the P/E-gate. (a) The angles of rotation of the head of the small subunit are plotted against *R_1_*. For comparison, the values for the X-ray structure (2WRI/2WRJ), TI^PRE^s (TI^PRE(2tRNAs)^, TI^PRE(Fus)^ and TI^PRE(GDPCP 1/2)^), TI^POST^s (TI^POST(2tRNAs)^, TI^POST(Fus1)^ and TI^POST(Fus2)^) were plotted with triangles and circles, respectively. TI^POST^s are plotted at angle = −10.0° because they were outside the range of graph. (b) The widths of the P/E-gate are plotted against *R_1_*. The width of the P/E-gate was defined as the distance between the center of mass of A790, and the center of mass of G1338 and A1339. For comparison, the widths for the X-ray structure (2WRI/2WRJ), TI^PRE^s and TI^POST^s were plotted with triangles and circles, respectively. The values from the first and second “r-translocation” simulations are plotted with thick and thin lines, respectively.

Fig. 4(a) shows that the head rotated clockwise as *R_1_* went from 18 to 22 Å (from the POST to INT state), while the head rotated anticlockwise as *R_1_* went from 31 to 34 Å (from the INT to PRE state). The timing of the large change of the rotation of the head at *R_1_* = ∼18–22 Å and ∼31–34 Å roughly corresponded to the two free-energy barriers. Moreover, the timing of the large movements of tRNAs at *R_1_* = ∼18–22 Å (Fig. 3) corresponded to the free-energy barrier at *R_1_* = ∼19–22 Å (Fig. 2). Therefore, these free-energy barriers were possibly produced by the coupled motion of head rotation and tRNA translocation.

X-ray crystallography [11] and cryo-EM experiments [13] [15] [17] have observed a very large “head-swivel” in several of the ribosome-tRNA-EFG complexes. Atomic models constructed from EMD-5775 [17] and EMD-1799 [15] and an X-ray structure (PDB code: 4KD8/4KD9) [11] of the ribosome-tRNA-EFG complexes in the intermediate state, referred to as post-translocational intermediate TI^POST((2tRNAs)^, TI^POST(Fus1)^ and TI^POST(Fus2)^ respectively in this study showed the large head-swivel. (TI^POST(Fus1)^ is referred to as TI^POST^ in the literature of Ratje et al. [15]) The rotation of the head is considered to facilitate the movement of the mRNA and anticodons of tRNAs from the A and P sites toward the P and E sites [2] [13] [15]. By superposing these structures on the initial structure, the head angles of TI^POST(2tRNAs)^, TI^POST(Fus1)^ and TI^POST(Fus2)^ were measured to be −17.7°, −22.8° and −17.5°, respectively (Table 2). As for TI^POST(2tRNAs)^ and TI^POST(Fus2)^, *R_1_* (the ratchet angle) were estimated to be 33.1 Å (5.7°) and 37.8 Å (6.6°), respectively (Table 2). These high values of *R_1_* and the ratchet angle do not match these structures which are supposed to be close to the POST state. A similar phenomenon has been reported in the literature of by Agirrezabala et al. [51], and it is considered that the structural change from the ribosome in the PRE state to TI^POST(Fus1)^ is not a rigid body-like motion [51]. (Hereafter, TI^POST((2tRNAs)^, TI^POST(Fus1)^ and TI^POST(Fus2)^ are simply referred to as TI^POST^ unless a distinction between them is needed.)

**Table 2:** The structural analysis of atomic models of the translocational ribosome.

Structure	*R_1_*	The angle of head	The width of the P/E-gate	The angle of ratchet	The state of tRNA	PDB code (EMD code) resolution
TI^PRE(2tRNAs)^	41.8 Å	−9.8°	23.9 Å	9.0°	P/E	3J5X/3J5W
					A/P*	(EMD-5800).
						7.6 Å [16].
TI^PRE(Fus)^	32.2 Å	−6.5°	25.4 Å	6.8°	P/E	2XSY/2XTG
						(EMD-1798)
						7.8 Å [15]
TI^PRE(GDPCP1)^	27.9 Å	−5.2°	24.2 Å	6.8°	P/E	4BTC/4BTD
						2.95 Å [8]
TI^PRE(GDPCP2)^	27.9 Å	−5.5°	24.0 Å	6.8°	P/E	4JUW/4JUX
						2.86 Å [9]
TI^POST(2tRNAs)^	33.1 Å	−22.8°	31.2 Å	5.7°	ap/P^1^	3J5N/3J5O
					pe/E	(EMD-5775)
						6.8 Å [17]
TI^POST(Fus1)^	37.8 Å	−17.5°	32.8 Å	6.6°	pe/E	2XUY/2XUX
						(EMD-1799)
						7.6 Å [15]
TI^POST(Fus2)^	20.0 Å	−17.7°	30.3 Å	3.4°	pe*/E^2^	4KD8/4KD9
						3.5 Å [11]
Initial	0.0 Å	−1.6°	24.1 Å	0.0°	E/E	2WRI/2WRJ
					P/P	3.6 Å [10]

1 In TI^POST(2tRNAs)^, two tRNAs are present in ap/P and pe/E hybrid states, where the lowercase and uppercase letters indicate tRNA contacts on the small and large subunit, respectively, in the following order: 30S head (a-site or p-site), 30S body/platform (p-site or e-site) and 50S subunit (P-site or E-site).

2 In TI^POST(Fus2)^, a single tRNA is present in a pe*/E hybrid state, where e* in pe*/E means that the anticodon stem-loop (ASL) of the tRNA lies between the p- and e-sites of the 30S body/platform.

TI^PRE(2tRNAs)^ consists of the ribosome, two tRNAs, mRNA, EF-G, GDP and viomycin. Each TI^PRE(Fus)^, TI^POST(Fus1)^ and TI^POST(Fus2)^ consists of the ribosome, a single tRNA, mRNA, EF-G and fusidic acid (Fus). Each TI^PRE(GDPCP1)^ and TI^PRE(GDPCP2)^ consists of the ribosome, a single tRNA, mRNA, EF-G, GDPCP. TI^POST(2tRNAs)^ consists of the ribosome, two tRNAs, mRNA, EF-G, GDP and Fus.

For 3J5X/3J5W and 3J5N/3J5O (*E. coli* ribosome), their superpositions on 2WRI/2WRI (*Thermus thermophiles* ribosome) were carried out using Chimera software's MatchMaker tool [73].

These results show the order of the direction of the rotation of the head observed in the “r-translocation” simulation was opposite to that in ordinary translocation.

### The P/E-gate opening and closing

It would be natural to assume that the atoms in the head region which directly interact with tRNAs are responsible for r-translocation. In this study, to understand which parts of the head region control r-translocation, we paid attention to a loop containing 16S rRNA nucleotides G1338 and A1339. This loop forms a gate with a 16S rRNA nucleotide A790 in the body region [2]. Hereafter, this gate which is located between the P and E-sites in the X-ray structure will be referred to as the P/E-gate, and the G1338-A1339 loop as the P/E-loop. Mutational studies have shown that the P/E-loop, especially A1339, is critical for translation *in vivo* [52] [53].

**Figure 5:**
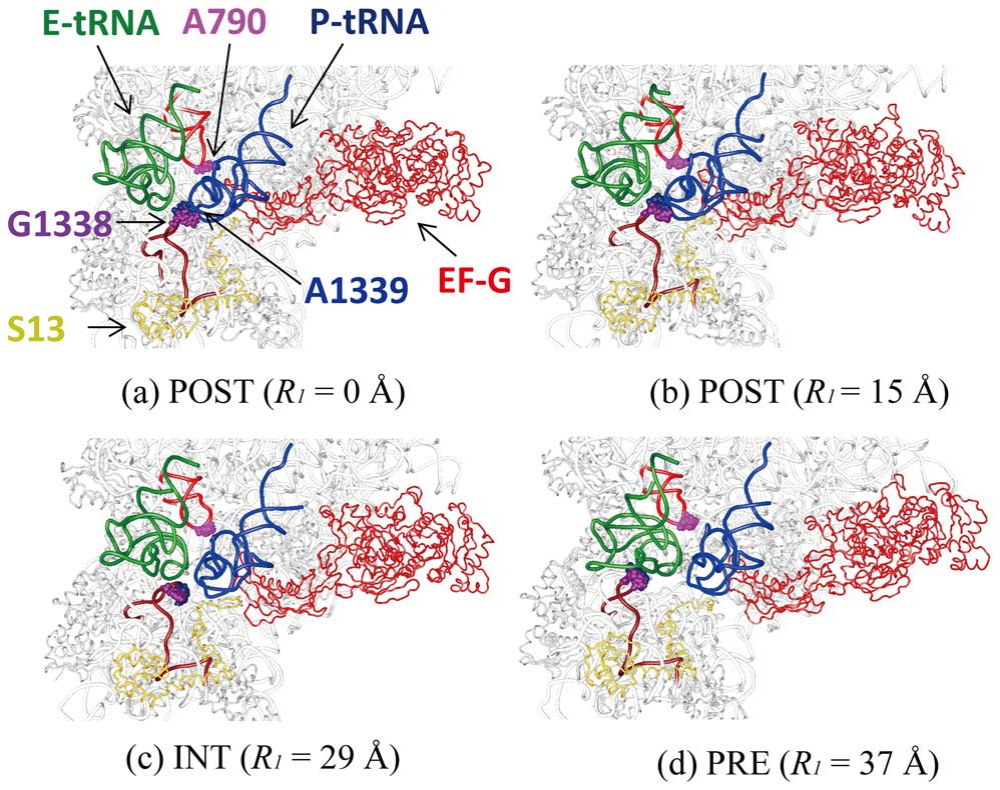
Conformational changes around the P/E-gate and the tRNAs in the POST, INT and PRE states. Snapshots of the conformations around the P/E-gate and the tRNAs on the small subunit in the (a) POST (*R_1_* = 0 Å), (b) POST (*R_1_* = 15 Å), (c) INT (*R_1_* = 29 Å) and (d) PRE (*R_1_* = 37 Å) states in the first “r-translocation” simulation are shown. The atoms of G1338, A1339 and A790, of the P/E-gate are depicted as space-filling models in purple, dark blue and pink, respectively. The loop regions of the 16S nucleotides A780-G799 and C1328-G1347 are depicted as wire models in red and brown, respectively. Protein S13 is also shown in yellow as a reference.

Fig. 4(b) shows that the P/E-gate moves from the closed to open position when r-translocation proceeds. The minimum width of the P/E-gate was 22 Å in the POST state at *R_1_* = ∼14–18 Å. As *R_1_* = 15 Å is the position in the lowest free-energy state, closing the gate should stabilize the ribosome and lock the tRNAs in their classical states. The P/E-gate was open wide in the PRE state, where the width was more than 30 Å. This width would be wide enough to allow the ASL of the E-tRNA to move from the E- to the P-site. Fig. 5 shows that the P/E-loop was on the exit side of the tRNA in the PRE state (*R_1_* = 37 Å), and it was deformed when the tRNA moved over the P/E-gate in the INT state (*R_1_* = 29 Å). The unfavorable interaction between the P/E-loop and the E-tRNA may contribute to the high free-energy barrier between the POST and INT states (Fig. 2). Thus, when r-translocation proceeds, the E-tRNA passes through the P/E-gate by overcoming the free-energy barrier between the POST and INT states, and then reaches the P/E site assisted by the opened P/E gate.

### The conformational changes of EF-G

EF-G binds to the intersubunit space on the A-site side of the ribosome and stabilizes the hybrid state of tRNA and the rotated state of the ribosome [4] [12] [13] [14]. Domains I (GTP-binding core domain G and insert subdomain G′) and V of EF-G interact primarily with the large subunit, while domains II, III, and IV interact primarily with the small subunit [10] [12] [13] [14]. Upon binding to the ribosome, EF-G undergoes large-scale conformational changes, where the tip of domain IV moves as far as ∼40 Å from its original position to the decoding center of the small subunit and overlaps the site occupied by the ASL of the A-tRNA [12]. Domain IV is considered to be functionally important for tRNA translocation [6] [54].

**Figure 6:**
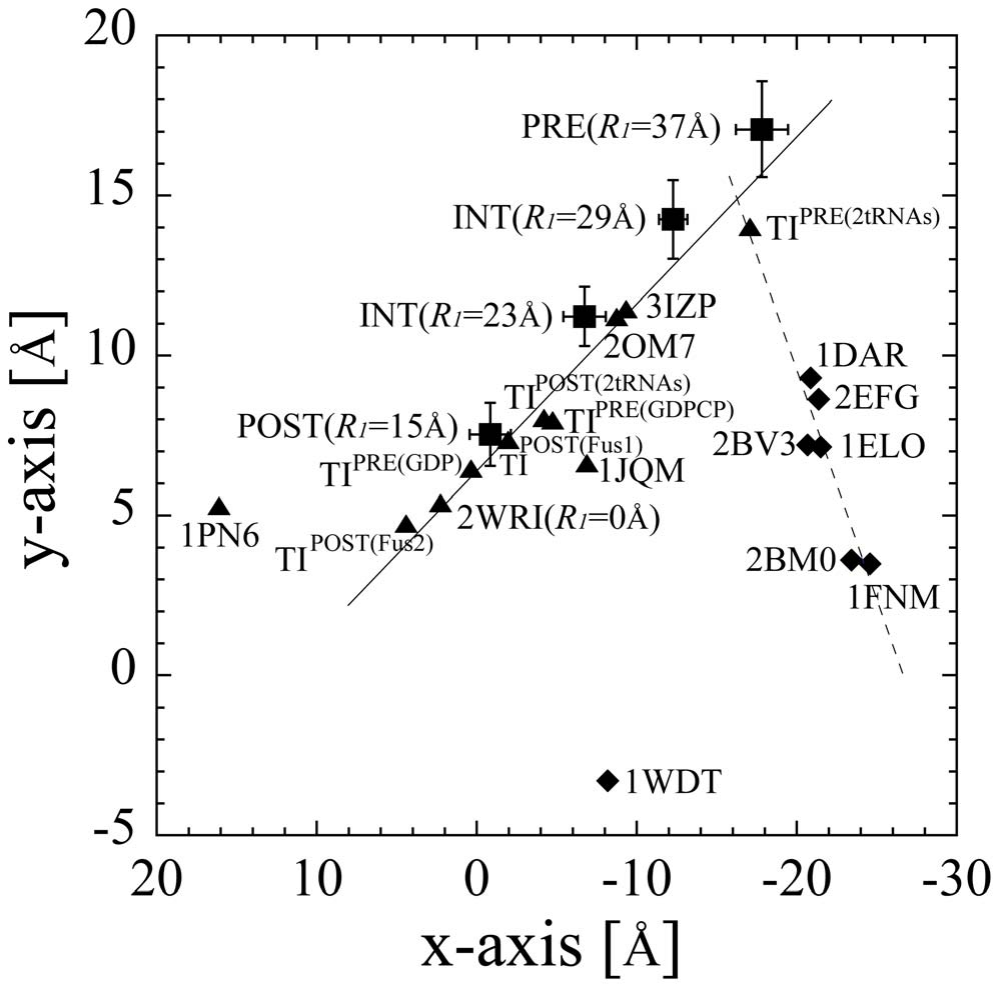
The movement of domain IV of EF-G. The positions of the center of mass of Gln500 in loop I and His 573 in loop II of domain IV of EF-G are plotted against a plane whose normal axis (z-axis) coincides with the unit vector of tRNA translocation, **e** *_tRNA_*, in Eq. (14). The y-axis was set to coincide with the axis of the ratchet-like movement. The x-axis was set as a cross product of the z-axis and y-axis. The origin is at the center of mass of the anticodon of P-tRNA. (The figure of these axes is shown in Fig. 6.) The positions of a single EF-G is shown with diamonds, and the positions of EF-G bound to the ribosome is shown with squares and circles. Structures of EF-G are from, simulated structures in the POST (*R_1_* = 15 Å), INT (*R_1_* = 23 and 29 Å) and PRE (*R_1_* = 37 Å) in the first “r-translocation” simulation, 3IZP (constructed by flexible fitting of a single EF-G into EMD-1365) [65], 1PN6 (constructed by rigid-body fitting of a single EF-G into EMD-1365) [12], 2OM7 (constructed from EMD-1315, GMPPNP-bound) [14], 1JQM (constructed from a cryo-EM map, fusidic acid and GDP-bound) [66], TI^PRE(2tRNAs)^ (3J5X/3J5W constructed from EMD-5800, GDP and viomycin-bound) [16], TI^PRE(Fus)^ (2XSY/2XTG constructed from EMD-1798, fusidic acid and GDP-bound) [15], TI^PRE(GDPCP1/2)^ (4BTC/4BTD and 4JUW/4JUX, GDPCP-bound) [8] [9], TI^POST(2tRNAs)^ (3J5N/3J5O constructed from EMD-5775, fusidic acid and GDP-bound) [17], TI^POST(Fus1)^ (2XUY/2XUX constructed from EMD-1799, fusidic acid and GDP-bound) [15], TI^POST(Fus2)^ (4KD8/4KD9, fusidic acid and GDP-bound) [11], 2WRI/2WRJ (X-ray structure at *R_1_* = 0 Å) [10], 1FNM (single EF-G mutant H573A, GDP-bound) [67], 2BM0 (single EF-G mutant T84A, GDP-bound) [68], 2BV3 (single EF-G mutant T84A, GDPNP-bound) [69], 2EFG (single EF-G wild-type, GDP-bound) [70], 1ELO (single EF-G wild-type, nucleotide-free) [71], 1DAR (single EF-G wild-type, GDP-bound) [72], 1WDT (single EF-2 homolog, GTP-bound) [14]. These structures of EF-G were superposed on the structure of EF-G in 2WRI/2WRJ (at *R_1_* = 0 Å) so as to minimize the structural differences in the main-chains of domains I and II (K4-L399). For 1WDT (single EF-2 homolog), Gln473 and His543 are used instead of Gln500 and His573, respectively. For 3J5X/3J5W and 3J5N/3J5O (*E. coli* EF-G), Gln508 and His584 are used instead of Gln500 and His573, respectively. The superpositions of 1WDT, 3J5X/3J5W and 3J5N/3J5O were carried out using Chimera software's MatchMaker tool [73]. The regression line for the positions of a single EF-G (except for 1WDT) is shown by broken line, and the regression line for the positions of EF-G bound to the ribosome (except for 1PN6) is shown by solid line.

To understand how the conformational change of domain IV of EF-G occurs during tRNA translocation, a variety of structures of EF-G determined by X-ray crystallography and cryo-EM were analyzed as shown in Fig. 6. These structures of EF-G were superposed on the structure of EF-G in 2WRI/2WRJ (at *R_1_* = 0 Å) so as to minimize the structural differences in the main-chains of domains I and II (K4-L399). Fig. 6 shows the position of the center of mass of Gln500 in loop I and His573 in loop II at the tip of domain IV, which directly interact with the P-tRNA in the X-ray structure [10]. Fig. 6 indicates that the conformations of EF-G in the free state and those in the ribosome-bound state during tRNA translocation are essentially different. Interestingly, the regression lines for EF-G bound and a single EF-G intersect at around the PRE state (*R_1_* = 37 Å) and TI^PRE(2tRNAs)^. This indicates that the conformational change in a single EF-G may cause it to bind to the ribosome in the PRE state. In principle, structures of 1PN6, 3IZP and the simulated structure in the PRE state should be the same as one another, because they were based on the same EM map (EMD-1365). However, 3IZP and the simulated structure in the INT state (*R_1_* = 23 Å) were located nearby. The difference between 3IZP and the simulated structure in the PRE state may be the result of different flexible fitting methods. 3IZP was constructed by fitting a single EF-G (PDB code: 1FNM) to the part of the EM map which corresponds to EF-G [35], while the structure in this study was constructed by fitting the ribosome-tRNAs-mRNA-EFG complex to the whole EM map. In contrast, 1PN6 is located far away from them. Probably this is because 1PN6 resulted from manual fitting using the domains of a single EF-G (PDB code: 1FNM) as rigid bodies into EMD-1365 with a low resolution of ∼11 Å.

**Figure 7:**
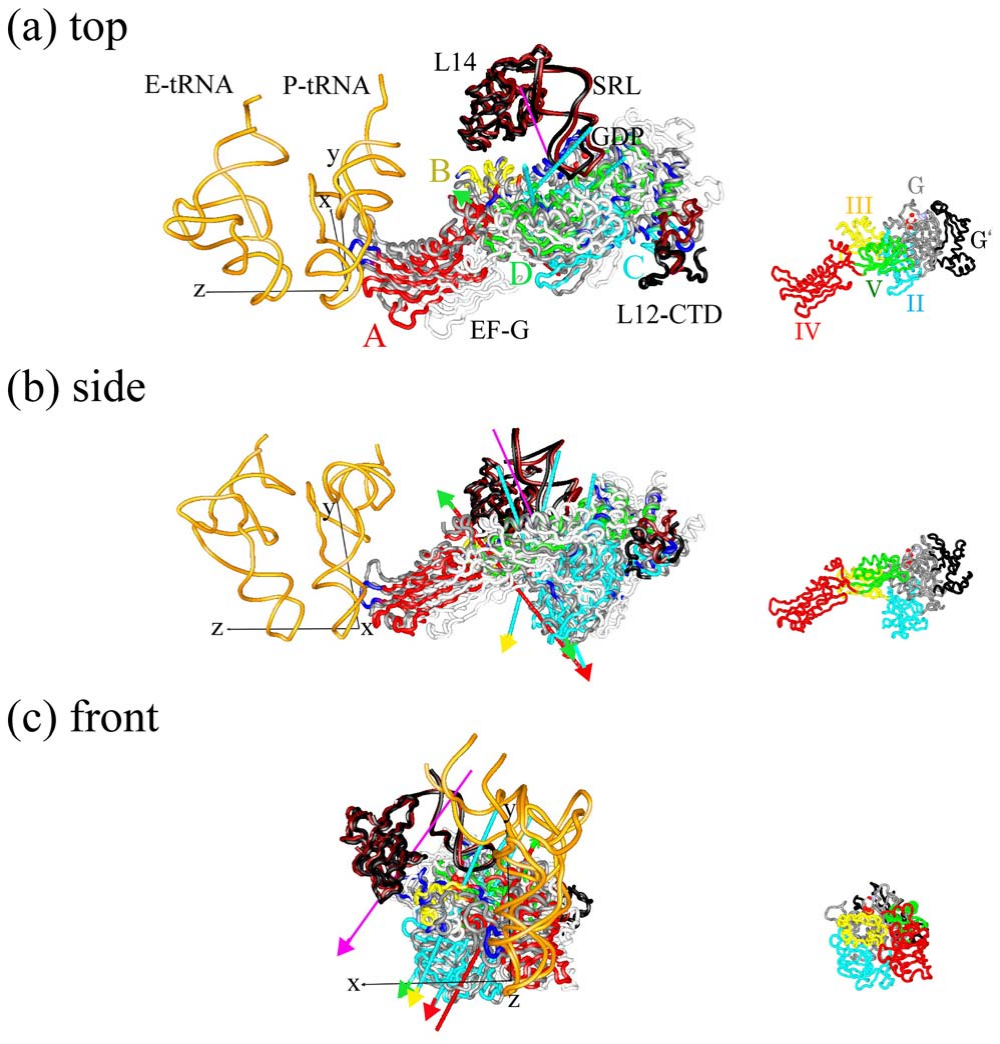
The internal and external motions of EF-G. The internal and external motions of EF-G shown from (a) side, (b) top and (c) front of EF-G. The structure of EF-G in the PRE state (*R_1_* = 37 Å) (in white) superposed on that in the POST state (*R_1_* = 15 Å) is depicted as a wire model in gray. The P- and E-tRNAs in the POST state are depicted as wire models in orange. The surrounding molecules, SRL (C2646-G2674), L14 and L12-CTD (L-chain, N69-P98) in the POST and PRE states are depicted as wire models in black and brown, respectively. GDP in the POST state is depicted as a space-filling model. Dynamic domains in the ribosome-bound EF-G for structures in the POST and PRE states were analyzed by DynDom3D program [49]. Each dynamic domain is colored: dynamic domain A (a large part of domain IV) in red, dynamic domain B (a large part of domain III) in yellow, dynamic domain C (a large part of domain II, and a part of domain G and subdomain G′) in thin blue and dynamic domain D (a large part of domain V and a part of domain G and subdomain G′) in green. The regions that were not assigned to a dynamic domain are shown in blue. The axis of dynamic domain X and Y is depicted as an arrow in color for dynamic domain X with a tip in color for dynamic domain Y. Dynamic domain X rotates anticlockwise around the axis of dynamic domains X and Y with respect to dynamic domain Y from the PRE to POST state. In the input parameter for the DynDom3D, the minimum ratio of external to internal displacement of 0.65 was used (default is 1.0). The ratio of internal to external displacement determines the acceptance criterion for a given domain pair. This lower value was required due to noise often seen from MD results [30]. The axis of the external motion is shown in pink. As a reference, x-axis, y-axis (**e**_ratchet_) and z-axis (**e**_tRNA_) are depicted as thin black lines at the anticodon of P-tRNA. The length of these axes (1 Å) is magnified by a factor of 50. EF-G with the same orientation as (a–c) is shown as a reference on the right side in small scale, where structural domains of G, G′, II, III, IV and V of EF-G are colored in gray, black, thin blue, yellow, red and green, respectively.

To understand what kinds of conformational changes in EF-G control the movement of domain IV, the conformational change between the structures in the lowest free-energy state in the POST state (*R_1_* = 15 Å) and in a local free-energy minima state in the PRE state (at *R_1_* = 37 Å) was decomposed into the internal and external motions. Fig. 7 shows that DynDom3D classified the internal dynamic domains into four parts: domain A–D. The axes of domains A and C, A and D, C and D show that EF-G undergoes the hinge-like motion around the axes of the internal dynamic domains so as to close the conformation when r-translocation proceeds. The angular magnitudes of rotation around the axes of domains A and C, A and D, and C and D, were estimated to be 28.2, 18.9 and 11.1°, respectively. The axis of domains B and C indicates that the space between domains III and G closes when r-translocation proceeds. The angular magnitude of rotation around the axis of domains B and C was estimated to be 15.6°. Moreover, the GTP binding region was located at the boundary between domains C and D. Therefore, the axis of domains A and B (closing motion between domains III and G from the POST to PRE state), C and D (twist motion in domain G) may be related to conformational changes within the GTP binding region in domain G and affect the affinity of inorganic phosphate (P_i_) produced by the GTP hydrolysis. Domains III has been implicated in catalyzing GTP hydrolysis and releasing P_i_ [14] [55].

The external motion of EF-G through the ribosome was interpreted as the result of a clockwise rotational movement by 13.4° around an axis passing near the sarcin-ricin loop (SRL) of the 23S rRNA, and a translational movement of EF-G by −1.9 Å along the axis when r-translocation proceeds. SRL is important for the binding of translation factors that hydrolyze GTP and is involved in triggering GTP cleavage [8] [9] [14]. This result that EF-G rotates around SRL is consistent with experimental data indicating that SRL is also crucial for anchoring EF-G on the ribosome during translocation [56] [16] [57]. The ratio of the magnitudes of the internal and external motion, 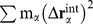
and 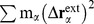, to the magnitude of the total motion, 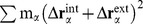, in domain IV and the whole structure of EF-G was 1.46: 0.25 and 0.83: 0.17, respectively. Therefore, the movement of domain IV of EF-G was mainly from the external motion. Fig. 7 shows that the C-terminal domain of L12 (L12-CTD) contacts subdomain G′. L12-CTD and subdomain G′ have also been implicated in catalyzing GTP hydrolysis and releasing P_i_ [10] [58] [59]. As the direction of the external motion at L12-CTD roughly coincides with the direction of the ratchet-like motion of the large subunit, L12-CTD would be the main contributor to the external motions of EF-G. The internal motion in domain IV, which comes from the hinge-bending motion, counteracts the external movement of domain IV.

## Discussion

### Model of r-translocation

**Figure 8:**
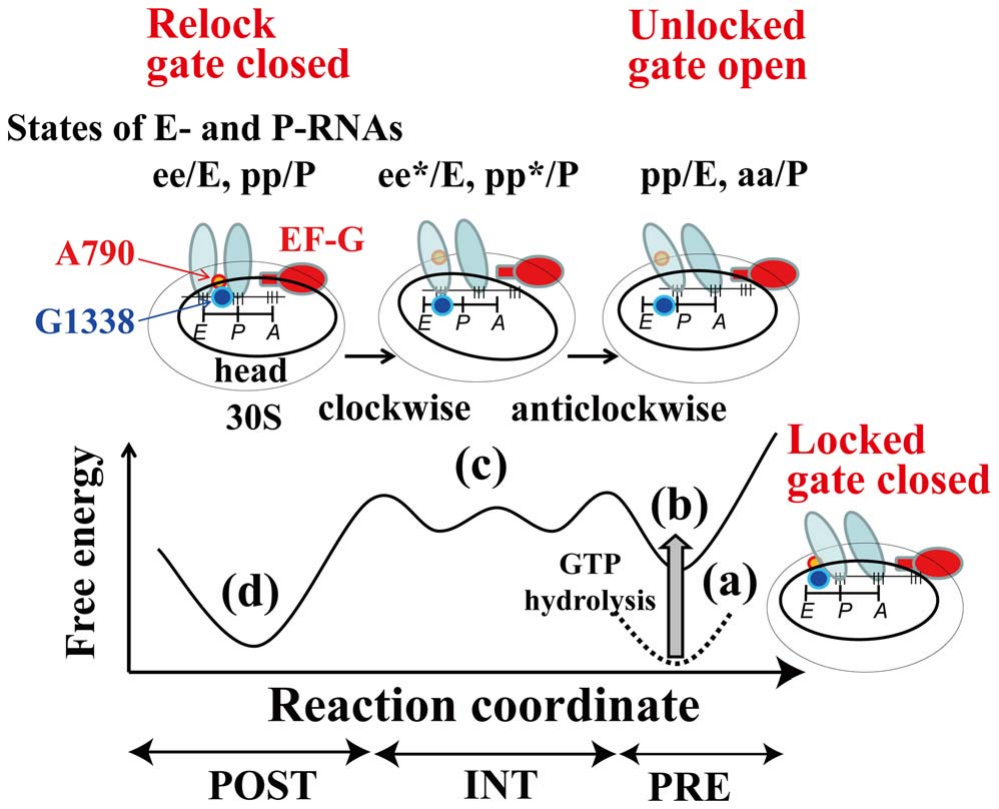
Model of r-translocation. Schematic representation of a model of r-translocation for (a) the ribosome in the PRE and locked states (tRNAs in the pp/E and aa/P states), (b) the ribosome in the PRE and unlocked states just after unlocking (tRNAs in the pp/E and aa/P states), (c) the ribosome in the INT and unlocked states (tRNAs in the ee*/E and pp*/P states) and (d) the ribosome in the POST and relocked states (tRNAs in the ee/E and pp/P states) are shown schematically. The large subunit of the ribosome is not shown for simplicity. (a) and (d) are in the locked and relocked states, respectively (closed state of the P/E-gate). In (a) and (d), the distance between A790 and G1338 of the P/E-gate is so narrow that the P/E-gate blocks the movement of the E-tRNA. (b) and (c) are in the unlocked state (open state of the P/E-gate). In (b) and (c), the distance between A790 and G1338 of the P/E-gate is wide enough to allow the E-tRNA to translocate from the E-stie to P-site.

We propose a model for r-translocation, based on the X-ray structure in the POST state and the cryo-EM maps of EMD-1363 and EMD-1365 as schematically shown in Fig. 8. In r-translocation, the ribosome goes up from the lowest free-energy minimum in the POST state (*R_1_* = ∼15 Å) through mainly two free-energy barriers between the POST and INT states (*R_1_* = ∼20 Å), and between the INT and PRE states (*R_1_* = ∼30 Å) (Fig. 8(c)). At the free-energy barrier between the POST and INT states, the clockwise rotation of the head from the POST to INT state would be coupled to the movement of the E-tRNA from the classical state by opening the P/E-gate. In this process, the anticodon of the E-tRNA and P-tRNA moved towards the P- and A-sites on the small subunit respectively (Fig. 3), while the clockwise rotation of the head worked to maintain the relative position between the head and the translocating tRNAs. Therefore, in the INT state, the two tRNAs were in the ee*/E and pp*/P states (Fig. 8(c)). (See the footnote of Table 2 for the meaning of the ee*/E and pp*/P states). At the free-energy barrier between the INT and PRE states, the anticlockwise rotation of the head from the INT to PRE state would be coupled to the location of the two tRNAs in the hybrid state over the opened P/E-gate (Fig. 8(b)). The clockwise rotation of the head from the POST to INT and the anti-clockwise rotation of the head from the INT to PRE may occur in the back-translocation facilitated by EF-4.

As the P/E-loop is a part of the head region, the movement of the P/E-loop is accompanied by the rotation of the head. Therefore, the rotation of the head and opening and closing of the P/E-gate would be strongly coupled. Consequently, tRNA and the P/E-gate may play the role of a pawl and a tooth of a gear (or head) respectively. The role of head as a gear would control the order of the rotation of the head according to the direction of tRNA translocation.

### Model of unlock and relock of the movement of tRNA

Although the transitions between the POST and PRE states in ordinary and reverse translocations would be different, it would be reasonable to assume that the structures in the POST (or PRE state) in those translocations are similar to each other. In ordinary translocation, upon GTP hydrolysis on EF-G in the PRE state, tRNAs are unlocked to translocate from the P/E- and A/P-sites in the hybrid state to the E/E- and P/P-sites in the classical state respectively [6], [13]. Then when tRNAs move in the classical state, tRNAs are relocked not to go back to the hybrid state [23].

In the “r-translocation” simulation, the P/E-gate was open in the PRE state. In fact, there are atomic models with a closed gate in the PRE state referred to as the pre-translocational intermediate TI^PRE^ [8] [9] [15] [16] (Table 2). (In Table 2, four different TI^PRE^s (TI^PRE(2tRNAs)^ [16], TI^PRE(Fus)^ [15], TI^PRE(GDPCP1)^ [8] and TI^PRE(GDPCP2)^ [9] are listed. However, they are simply referred to as TI^PRE^, unless a distinction between them is needed.) In TI^PRE^, the P/E-loop is on the exit side of the E-tRNA and the P/E-gate is closed (the width of the P/E gate is ∼24–25 Å). This indicates that the P/E-gate contributes to blocking the movement of tRNA. The angle of the head and *R_1_* were estimated to be ∼−5–10° and ∼28–42 Å, respectively (Fig. 4(a)). Therefore TI^PRE^ correspond to the locked state of the movement of tRNA-mRNA, and may fluctuate around *R_1_* = ∼28–42 Å in a local free-energy minima state. In contrast to the “locked” state in TI^PRE^, the structure with the gate open wide in the PRE state observed in the “r-translocation” simulation is thought to represent a state just after the ribosome is unlocked by the energy of GTP hydrolysis (Fig. 8(b)).

“Unlocking” would correspond to the opening of the P/E-gate and the rotation of the head. Once TI^PRE^ is “unlocked” at *R_1_* = ∼30 Å, the A/P-tRNA would translocate from the A/P-state (*R_1_* = ∼30 Å) to P/P-state (*R_1_* = ∼15 Å) spontaneously. If the free-energy barrier of ∼8–10 kcal/mol at *R_1_* = ∼30 Å is the result of the GTP hydrolysis for TI^PRE^, the P/E-gate closed state of TI^PRE^ would have a free-energy of ∼1–3 kcal/mol (free energy of GTP hydrolysis is −7.3 kcal/mol under standard conditions [60]). In this case, TI^PRE^ would be as stable as the post-translocational ribosome (Fig. 8(a)). The most closed state of the P/E-gate was observed at the position of the lowest free-energy (*R_1_* = ∼15 Å). In ordinary translocation, “relock” would correspond to the closing of the P/E-gate (at *R_1_* = ∼15 Å) to fix the tRNAs in the new classical state and block the reverse movement of tRNA-mRNA (Fig. 8(d)).

However, it should be noted that the free-energy cannot be decomposed into the components of conformational changes like the ratchet, head, PE-gate and tRNA movements as the free-energy is in principle calculated from a partition function in the system where these movements cannot be exactly separable. Not only one component but all components which are dynamically coupled with each other would contribute to the low free-energy minima and high free-energy barriers. Possibly, the mechanism of the “lock” and “relock” should be regarded as the result of the total contribution from these complicated movements.

### The role of the conformational changes in EF-G

The maintenance of the interaction between the A/P-tRNA and domain IV of EF-G observed in the “r-translocation” simulation (Fig. 3) would be the same as in the ordinary translocation [10]. Assuming that the conformational changes in EF-G are independent of the possible irreversible movement of the head region, the conformational changes in EF-G observed in the “r-translocation” simulation would be reversible in the ordinary translocation. In this case, when the ordinary translocation proceeds, the opening motion of the space between domains III and G and twist motion in domain G (Fig. 7) would open the GTP binding region and facilitate the release of P_i_ after the GTP hydrolysis. Therefore, in the ordinary translocation, the internal hinge-bending motion may play two roles (1) in controlling the orientation of domain IV for the maintenance of its interaction with the A/P-tRNA to block the reverse movement of tRNA-mRNA, and (2) in releasing P_i_ after the GTP hydrolysis. Moreover, the external rotational motion triggered by L12 utilizing the back ratchet-like movement would contribute to the extension of domain IV of EF-G towards the A/P-tRNA.

### Comparison with the experimental data

The dynamics of the large head-swivel suggested by EMD-1343 [13], TI^POST^ [11] [15] [17] has been observed by FRET [21]. The experiment showed that the head-swivel was fast, in ∼80 s^−1^, while the following back head-swivel and back ratchet-like movement were slow, in ∼10 s^−1^ and ∼4.5 s^−1^ [21]. The observation that the head-swivel was fast and the back head-swivel was slow indicates that TI^POST^ may be in a local free-energy minima state and there may be a high free-energy barrier between the state of TI^POST^ and the POST state. In TI^POST^, the P/E-gate opens, where the width of the P/E gate was ∼30–33 Å (Table 2 and Fig. 4(b)). A790 is located at the body side of the P/E-tRNA, but the P/E-loop is still at the exit side of the P/E-tRNA. Therefore, in the ordinary translocation, as the head rotates clockwise in order to go back to the original position in the POST state, the P/E-gate should be moved from the exit side of the P/E-tRNA to between the E- and P-sites on the small subunit. This movement of the P/E-gate over the tRNA may be the rate-limiting kinetic step observed in the FRET experiment. The possible existence of the high free-energy barrier for the back head-swiveling due to conformational rearrangement between the tRNAs and the head has also been discussed in the literature of Agirrezabala and Frank [61].

Under special conditions (for example, the addition of deacylated tRNAs cognate to the codon at the E-stie), it has been shown that tRNA-mRNA can spontaneously move backward from the POST to PRE state in the absence of EF-G [26] [27] or in the presence of EF-G, GTP and antibiotics [28]. However, a large anticlockwise rotational movement of the head by ∼15° as observed in TI^POST^, was not observed between the POST and PRE states by the time-resolved EM experiment of reverse translocation [26]. This may be because EF-G was not included in the experiment. (EMD-1524 (ribosome bound to EF-4 in the PRE state) [25] did not show the large head-swivel either.) The movement of the head in the reverse translocation may be different from that in the ordinary translocation, as was shown in “r-translocation” simulation.

### Accuracy and limitations of the computational simulations

First, we would like to point out the arbitrary nature of the choice of the umbrella sampling variables and the strength of their restrains during the umbrella sampling simulations Although 50 umbrella sampling variables and positional restraints of 1.0 kcal/mol/Å^2^ were used in this study, less umbrella sampling variables and less positional restraints could have been better in some regions (see Text S5 “The choice of the umbrella sampling variables and the strength of their restraints” and Fig. S7). However, the best choice of them *a priori* is difficult because the free-energy landscape is unknown before the calculation. As the path-search algorithm was used to adjust the strength of the restraints for the tRNA movement in this study, it may also be useful to apply the path-search algorithm to adjust the strength of the restraints for the ribosomal movement.

Second, in this study, the AMBER parm99SB force-field for the monovalent K^+^ and Cl^−^ ions was used although the force-field parameters for these ions can cause artifacts in MD simulations due to the strong ion-ion interactions [62] [63]. To estimate the influence of the force-field for K^+^ and Cl^−^ ions on the free-energy landscape, another MD simulation was carried out using a corrected force-field which was developed by Joung and Cheatham [64]. The results showed that the influence due to different ion force-field was not significant in this study (see Text S6 “The effect of different ion force-fields on the free-energy landscape” and Fig. S2(h)).

Finally, in this study, MD simulations were carried out for ∼1 µs to obtain the free-energy profile for r-translocation. First, four free-energy profiles for the “classical-tRNA” simulation were compared (Fig. S2(a)–(d)). The lowest free-energy profile for the “classical tRNA” simulation (Fig. S2(c) or S2(e)) was expected to give the upper limit of the free-energy for r-translocation because the ratchet-movement without tRNA translocation in the “classical tRNA” simulation is not natural. However, it should be noted that two main free-energy stable states were observed at *R_1_* = ∼15 and ∼35 Å in the “classical tRNA” simulation, indicating that these stable or more stable states would exist at these positions for r-translocation and indeed these stable states were found to coincide with the distinctive movement of the P/E-gate and the head rotation in the “r-translocation” simulation. Therefore, the existence of these stable states at *R_1_* = ∼15 and ∼35 Å is thought to be sufficiently reliable.

Two paths, one for the “classical tRNA” simulation and the other for the “r-translocation” simulation, were obtained from the umbrella sampling simulations. From the viewpoint of the 50 umbrella coordinates of which each path consists, these two paths were not globally very different from each other. (Their inner product at **R** ^f^ was nearly 1, 0.957 (Table 1)). This is because the fitting procedures for the 50 constitute molecules in the system were substantially the same for both simulations except for the treatment of the tRNAs. Therefore, the difference in the two free-energy profiles is thought to be mainly from the difference in the movement of the tRNAs and the accompanying movements of the P/E-gate, head rotation and EF-G. These movements reduced the high free-energy at the INT state in the “classical tRNA” simulation. The path in the “classical tRNA” simulation produced a rather smooth free-energy curve, indicating that the data sampling along this path would be sufficient to produce a reliable free-energy profile. In contrast, the paths in the two “r-translocation” simulations between *R_1_* = ∼19 and ∼33 Å in the INT state produced rather rugged free-energy curves. As the ribosome is a very complicated system, there would be many paths in the INT state during tRNA translocation and two paths in the INT state may not be sufficient to produce an accurate free-energy. To improve the accuracy of the free-energy in the INT state, more sampling for other paths, more cryo-EM data and experimental data in the INT state would be required; however, if so much data were available, carrying out such large-scale simulations would not be feasible due to the current computational limitations. However, it is believed that the coupled motion of the head rotation and tRNA translocation during the ratchet-like movement in the ribosome would be the main contributor to the free-energy profile in the other possible intermediate states.

## Conclusions

Using EM-fitting and molecular dynamics simulations based on the structures observed by cryo-EM and X-ray crystallography, we obtained the free energy landscape of reverse translocation. Our simulation showed that reverse translocation passes through a series of conformational changes such as a large conformational change of the ratchet-like movement, the rotational movement of the head, the opening and closing of the P/E-gate and the internal hinge-bending and external movements of EF-G. In future, incorporation of cryo-EM structures and biochemical experimental data related to the translocational ribosomes into computational simulation studies like this study will give a more detailed understanding of the whole reaction of translocation.

### Available atomic models of the translocational ribosome

The atomic models of the ribosome-tRNAs-mRNA-EFG complex: (1) an initial structure at *R_1_* = 0 Å (R0.pdb), (2) a structure fitted into EMD-1365 at *R_1_* = ∼30 Å in the “classical-tRNA” simulation (R30_fitted1365.pdb), (3) a transitional structure at *R_1_* = ∼26 Å (R26.pdb) and (4) a structure fitted into EMD-1363 at *R_1_* = ∼43 Å in the “r-translocation” simulation (R43_fitted1363.pdb), are available from PLOS ONE. As for structures (2) and (4), these structures were fitted into the actual EMD-1365 and EMD-1363 (fitted1365.pdb and fitted1363.pdb respectively). The details of these structures are given in Text S7, “Structures of the translocational ribosome obtained using molecular dynamics simulations and cryo-EM density maps”.

## Supporting Information

Figure S1 **Structural analysis in the EM-fitting simulation.** The average values of (a) the correlation coefficient, (b) the number of residues forming secondary structures of helices (α- and 3_10_- helices) and β-sheets in ribosomal proteins and EF-G during the umbrella sampling simulations, (c) the Ramachandran outlier indicator and (d) the first reaction coordinate of *R_1_* are plotted against the window number in the “classical tRNA” and the first “r-translocation” simulations. The number of residues forming secondary structures was analyzed using software called STRIDE [74]. The Ramachandran outlier indicator was analyzed using software called RAMPAGE [75]. The indicators of the X-ray structure of 2WRI/2WRJ and the initial (energy-minimized) structures were 10.0% and 3.3%, respectively. The indexes (a) with crosses, (e) with triangles and (f) with open and closed circles correspond to those in Fig. S2. *R_1_* in Fig. S1(d) was used in the same way as in Fig. S2.
(TIF)

Figure S2 **The free-energy landscapes in the “classical tRNA” simulation.** The free-energy landscapes, calculated according to Eq (15), are plotted against their first reaction cooridnates, *R_1_*, for cases: (a) the INT state of EMD-1365 (black line), (b) the INT state switching EMD-1363 to EMD-1365 at the 15-th window (*R_1_* = ∼14 Å) (dotted green line), (c) the INT state switching EMD-1363 to EMD-1365 at the 21-st windows (*R_1_* = ∼18 Å) (dotted red line), (d) the INT state switching EMD-1363 to EMD-1365 at the 26-th window (*R_1_* = ∼22 Å) (dotted black line), (e) “classical tRNA” (blue line and broken black line), (f) the first “r-translocation” (the same as Fig. 2(a), thick red line), (g) the second “r-translocation” (the same as Fig. 2(a), thin purple line) and (h) the simulation using a corrected force-field for K^+^ and Cl^−^ ions (dotted purple line) [64]. The lowest free-energy was set at zero for each case. It should be noted that the free-energy landscapes from *R_1_* = 0 to 15 Å are slightly different from one another even though the data used were the same because the reaction coordinates for each simulation are slightly different from one another (see the definition of R_ratchet_^f^ in Table 1). For comparison, the free-energy landscape of (e) with the reaction coordinate used in (f) is shown with a broken black line.
(TIF)

Figure S3 **Comparison of the movement of the P-tRNA, E-tRNA, and EF-G in the “classical tRNA” and “r-translocation” simulations.** (a) The paths of the centers of mass of the anticodons of the P-tRNA and E-tRNA, and of domain IV (residues 481–603 and the C-terminal region 675–690) of EF-G, the second reaction coordinates of *R_2_*, are plotted against *R_1_*, for the “classical tRNA” and “r-translocation” with dotted and solid lines, respectively. The same reaction coordinates of *R_1_* and *R_2_* which were defined in the first “r-translocation” simulation (11. in Table 1) were used for both cases. (b) Shapshots of the conformations of the two tRNAs and EF-G in the PRE state (*R_1_* = 37 Å) observed in the “classical tRNA” and “r-translocation” simulations are depicted as wire models in blue and red, respectively. The conformation in the X-ray structure (2WRI/2WRJ, *R_1_* = 0 Å) is depicted as a wire model in black.
(TIF)

Figure S4 **Prediction of a possible path for r-translocation from the two-dimensional free-energy landscapes in the classical, semi-hybrid and hybrid states.** The two-dimensional free-energy landscapes are plotted against *R_1_* and *R_2_* for cases: (a) classical, (b) semi-hybrid (c) hybrid states and (d) “r-translocation”. *R_2_* is the center of mass of the P-tRNA. The lowest free-energy was set at zero for each state, and the positions are shown with a cross at (*R_2_*, *R_1_*) = (1.8 Å, 15 Å), an open triangle at (−4.0 Å, 28 Å) and a open circle at (−11.0 Å, 39 Å) in the classical, semi-hybrid and hybrid states, respectively. The starting positions set by the SMD for the semi-hybrid and hybrid states are shown with a closed triangle at (−6.6 Å, 15 Å) and a closed circle at (−12.0 Å, 28 Å), respectively. The paths moved by the SMD from the classical to semi-hybrid state and from the semi-hybrid to hybrid state are depicted as dotted and broken lines, respectively. A possible path for r-translocation is depicted as a curved line connecting these lowest free-energy minima. The same reaction coordinate of *R_1_* which was defined in the “classical tRNA” simulation (6. in Table 1) was used for (a), (b) and (c), while the reaction coordinate of *R_1_* which was defined in the “r-translocation” simulation (11. in Table 1) was used for (d).
(TIF)

Figure S5 **Schematic representation for the “semi-hybrid” tRNA simulation.** The schematic representation for the (a) classical, (b) semi-hybrid and (c) hybrid states of tRNAs is shown. In the semi-hybrid state, the anticodons of the E- and P-tRNAs in the classical state move to between the E and P-sites and P and A-sites on the small subunit.
(TIF)

Figure S6 **The relationship between the first reaction coordinate,** ***R_1_*** **and the ratchet angle.** The relationship between the first reaction coordinate, *R_1_* and the ratchet angle in the first “r-translocation” simulation is shown. The maximum and minimum of the ratchet angle are shown in dotted and broken lines, respectively. For comparison, the values for TI^PRE^s and TI^POST^s (Table 2) were plotted with triangles and circles, respectively.
(TIF)

Figure S7 **The choice of the umbrella sampling variables and the strength of their restraints.** (a) The RMSFs of the centers of mass and (b) the mass-weighted RMSFs of the atoms of 16S rRNA in the small subunit, 23S rRNA in the large subunit, 25 proteins close to the center of mass of the ribosome and 25 proteins far from the center of mass of the ribosome are plotted against the protocol number. The protocol numbers 1–5 are for 50 restraints of 1.0 kcal/mol/Å^2^ (original), 25 restrains of 1.0 kcal/mol/Å^2^ for 25 close proteins, 50 restraints of 0.20 kcal/mol/Å^2^, 50 restrains of 0.04 kcal/mol/Å^2^ and no restraints on the umbrella sampling variables, respectively. The values for 16S rRNA, 23S rRNA, 25 close and far proteins are shown with triangles, inverted triangles, squares and circles, respectively. Open and closed marks are from the result of the simulations carried out at *R_1_* = ∼15 and 21 Å, respectively. (c) The average and RMSF of *R_1_* for protocols 1–5 are shown with open and closed circles for the simulations carried out at *R_1_* = ∼15 and 21 Å, respectively.
(TIF)

Text S1 **The free-energy landscape in the classical state of tRNA (“classical tRNA” simulation).**
(PDF)

Text S2 **Modeling of the semi-hybrid and hybrid states of tRNAs.**
(PDF)

Text S3 **The free-energy landscape in the semi-hybrid and hybrid states of tRNA (“semi-hybrid tRNA” and “hybrid tRNA” simulations).**
(PDF)

Text S4 **The verification of the “r-translocation” simulation.**
(PDF)

Text S5 **The choice of the umbrella sampling variables and the strength of their restraints.**
(PDF)

Text S6 **The effect of different ion force-fields on the free-energy landscape.**
(PDF)

Text S7 **Available atomic models of the translocational ribosome obtained using molecular dynamics simulations and cryo-EM density maps.**
(DOCX)
