# Supplementary figures and images for: Free-Energy Landscape of Reverse tRNA Translocation through the Ribosome Analyzed by Electron Microscopy Density Maps and Molecular Dynamics Simulations
Source: PLoS One. 2014 Jul 7;9(7):e101951. doi: 10.1371/journal.pone.0101951 (PMC4084982; doi:10.1371/journal.pone.0101951)

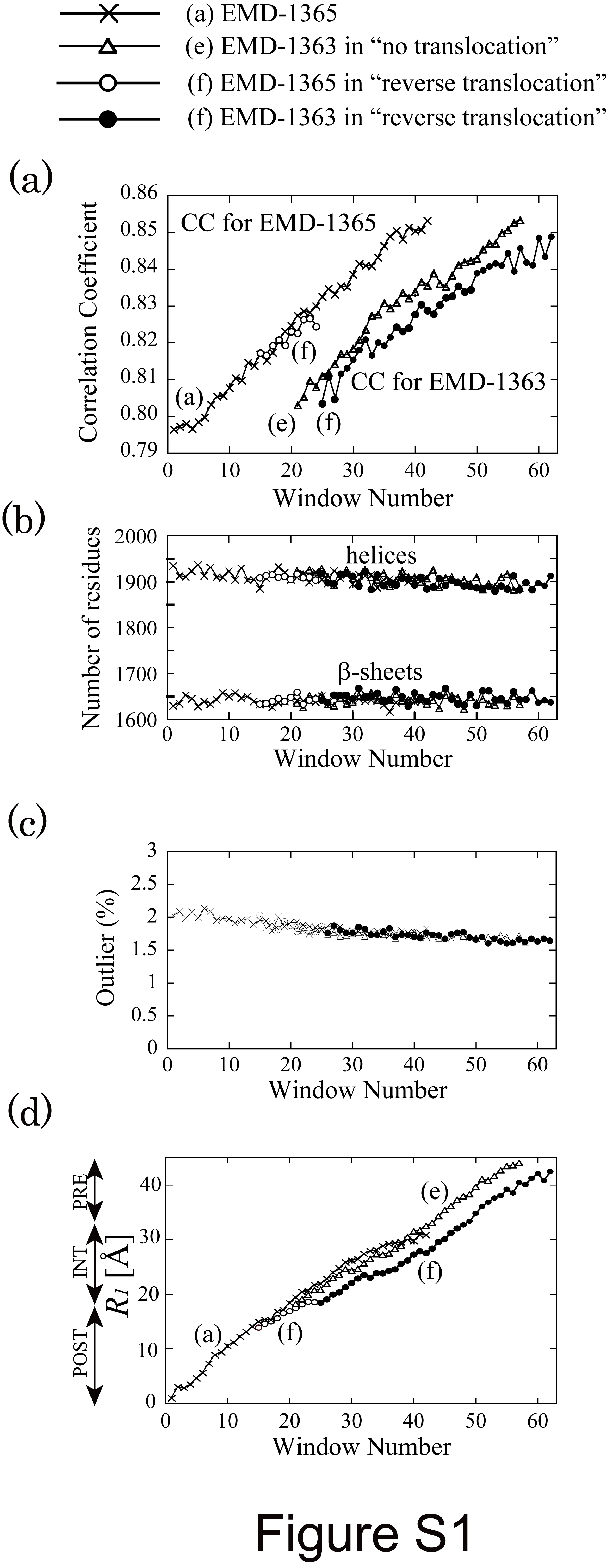

Supplement: Figure S1 — Structural analysis in the EM-fitting simulation. The average values of (a) the correlation coefficient, (b) the number of residues forming secondary structures of helices (α- and 310- helices) and β-sheets in ribosomal proteins and EF-G during the umbrella sampling simulations, (c) the Ramachandran outlier indicator and (d) the first reaction coordinate of R1 are plotted against the window number in the “classical tRNA” and the first “r-translocation” simulations. The number of residues forming secondary structures was analyzed using software called STRIDE [74]. The Ramachandran outlier indicator was analyzed using software called RAMPAGE [75]. The indicators of the X-ray structure of 2WRI/2WRJ and the initial (energy-minimized) structures were 10.0% and 3.3%, respectively. The indexes (a) with crosses, (e) with triangles and (f) with open and closed circles correspond to those in Fig. S2. R1 in Fig. S1(d) was used in the same way as in Fig. S2. (TIF) [file pone.0101951.s001.tif]

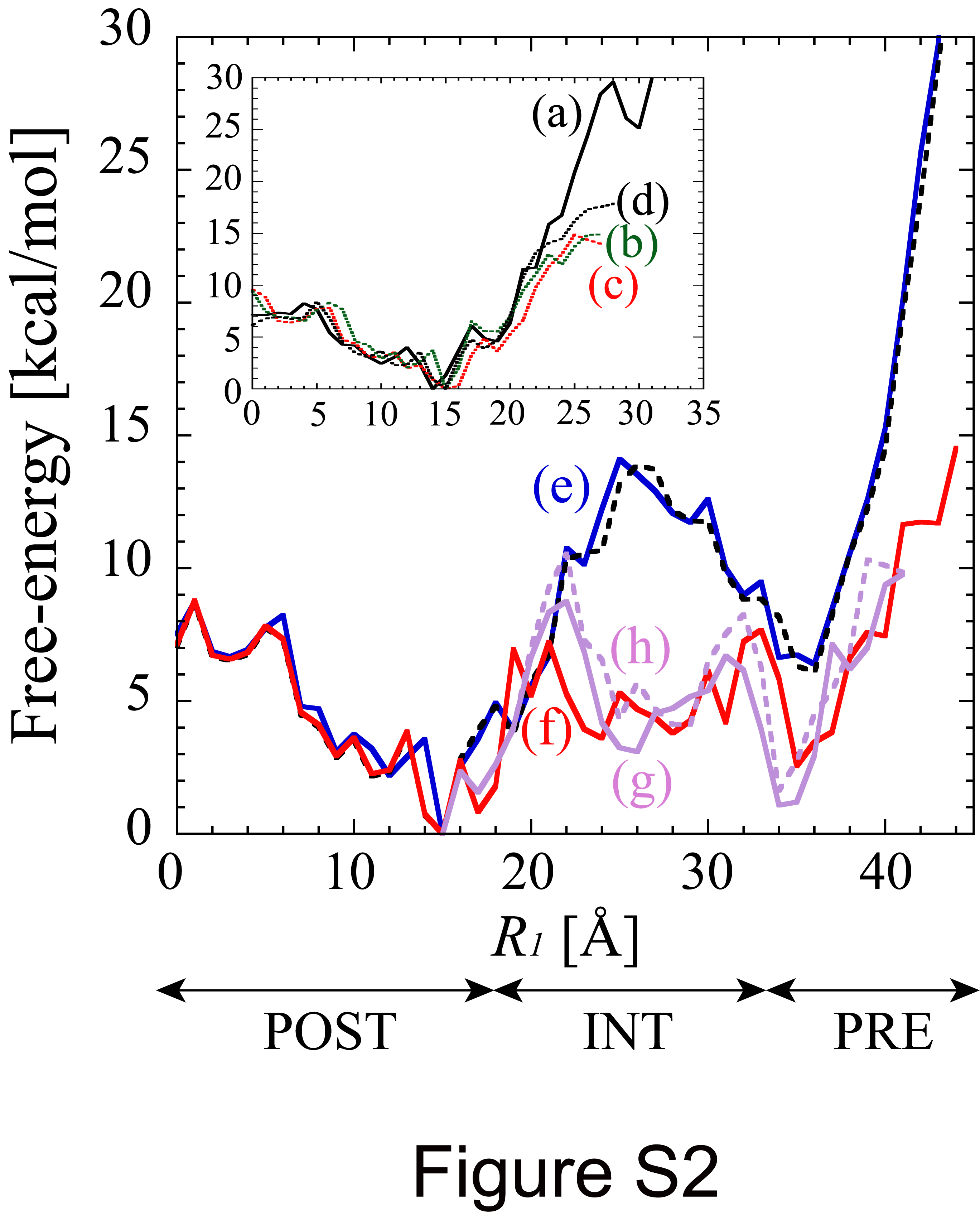

Supplement: Figure S2 — The free-energy landscapes in the “classical tRNA” simulation. The free-energy landscapes, calculated according to Eq (15), are plotted against their first reaction cooridnates, R1, for cases: (a) the INT state of EMD-1365 (black line), (b) the INT state switching EMD-1363 to EMD-1365 at the 15-th window (R1 = ∼14 Å) (dotted green line), (c) the INT state switching EMD-1363 to EMD-1365 at the 21-st windows (R1 = ∼18 Å) (dotted red line), (d) the INT state switching EMD-1363 to EMD-1365 at the 26-th window (R1 = ∼22 Å) (dotted black line), (e) “classical tRNA” (blue line and broken black line), (f) the first “r-translocation” (the same as Fig. 2(a), thick red line), (g) the second “r-translocation” (the same as Fig. 2(a), thin purple line) and (h) the simulation using a corrected force-field for K+ and Cl− ions (dotted purple line) [64]. The lowest free-energy was set at zero for each case. It should be noted that the free-energy landscapes from R1 = 0 to 15 Å are slightly different from one another even though the data used were the same because the reaction coordinates for each simulation are slightly different from one another (see the definition of Rratchet f in Table 1). For comparison, the free-energy landscape of (e) with the reaction coordinate used in (f) is shown with a broken black line. (TIF) [file pone.0101951.s002.tif]

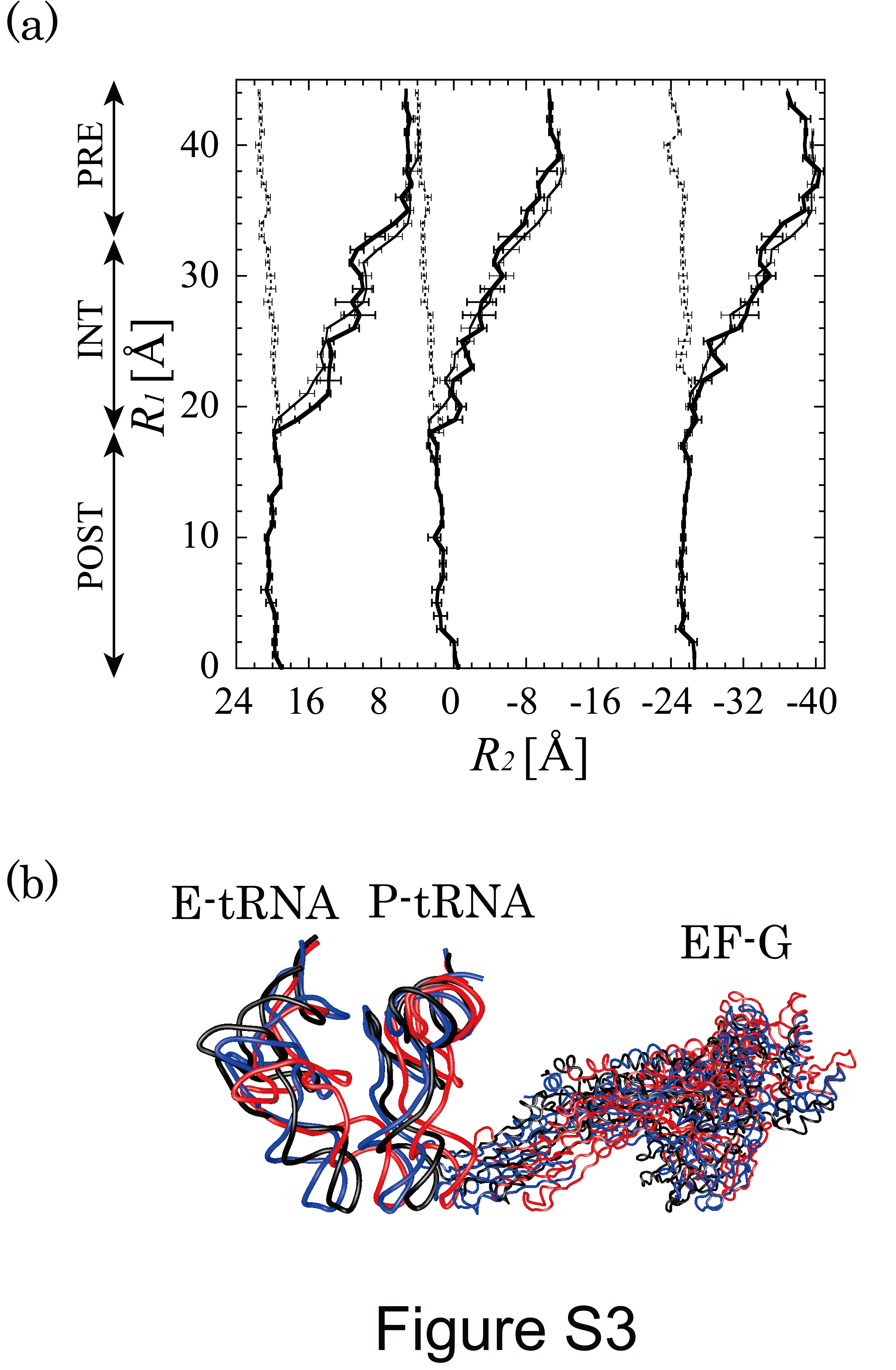

Supplement: Figure S3 — Comparison of the movement of the P-tRNA, E-tRNA, and EF-G in the “classical tRNA” and “r-translocation” simulations. (a) The paths of the centers of mass of the anticodons of the P-tRNA and E-tRNA, and of domain IV (residues 481–603 and the C-terminal region 675–690) of EF-G, the second reaction coordinates of R2, are plotted against R1, for the “classical tRNA” and “r-translocation” with dotted and solid lines, respectively. The same reaction coordinates of R1 and R2 which were defined in the first “r-translocation” simulation (11. in Table 1) were used for both cases. (b) Shapshots of the conformations of the two tRNAs and EF-G in the PRE state (R1 = 37 Å) observed in the “classical tRNA” and “r-translocation” simulations are depicted as wire models in blue and red, respectively. The conformation in the X-ray structure (2WRI/2WRJ, R1 = 0 Å) is depicted as a wire model in black. (TIF) [file pone.0101951.s003.tif]

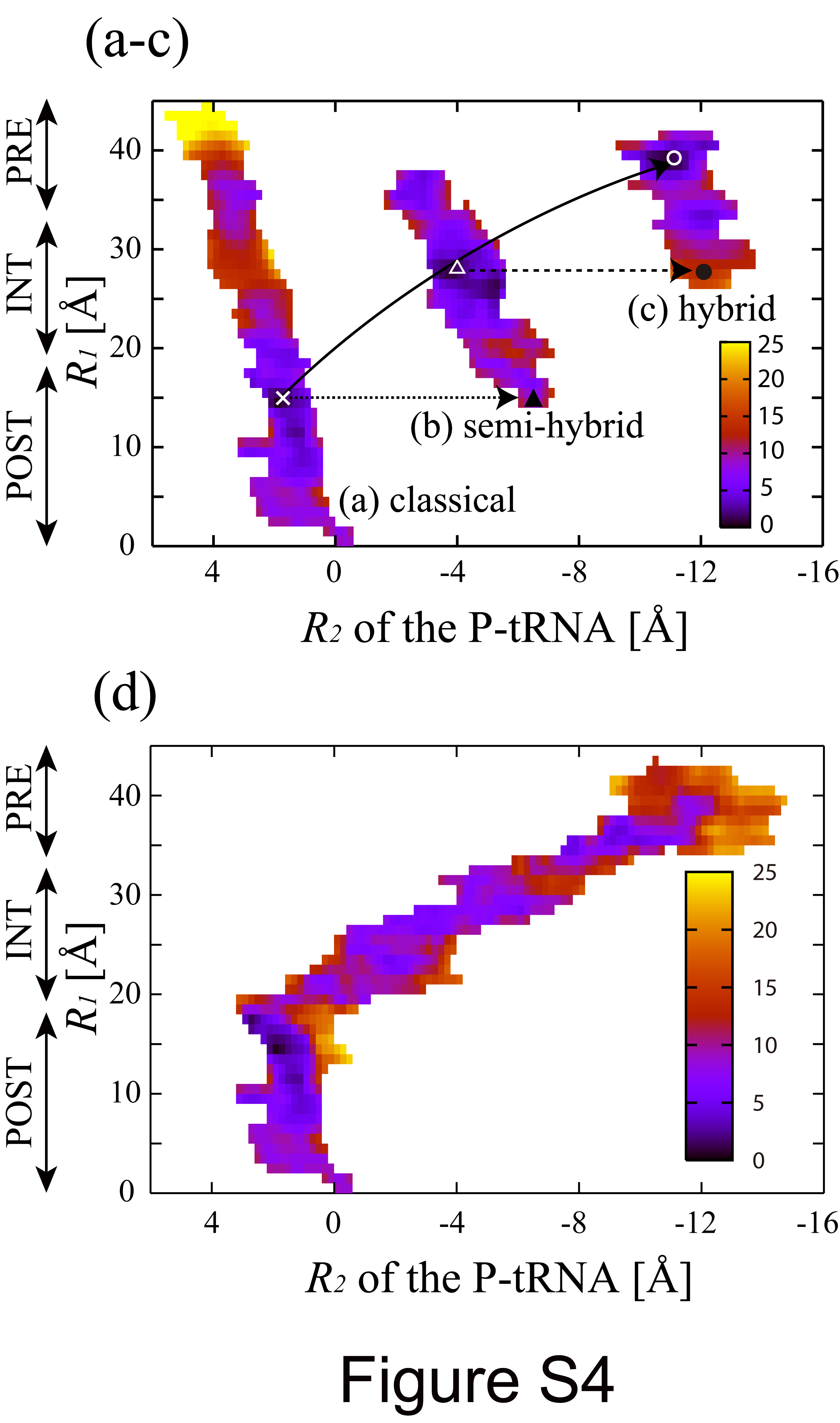

Supplement: Figure S4 — Prediction of a possible path for r-translocation from the two-dimensional free-energy landscapes in the classical, semi-hybrid and hybrid states. The two-dimensional free-energy landscapes are plotted against R1 and R2 for cases: (a) classical, (b) semi-hybrid (c) hybrid states and (d) “r-translocation”. R2 is the center of mass of the P-tRNA. The lowest free-energy was set at zero for each state, and the positions are shown with a cross at (R2, R1) = (1.8 Å, 15 Å), an open triangle at (−4.0 Å, 28 Å) and a open circle at (−11.0 Å, 39 Å) in the classical, semi-hybrid and hybrid states, respectively. The starting positions set by the SMD for the semi-hybrid and hybrid states are shown with a closed triangle at (−6.6 Å, 15 Å) and a closed circle at (−12.0 Å, 28 Å), respectively. The paths moved by the SMD from the classical to semi-hybrid state and from the semi-hybrid to hybrid state are depicted as dotted and broken lines, respectively. A possible path for r-translocation is depicted as a curved line connecting these lowest free-energy minima. The same reaction coordinate of R1 which was defined in the “classical tRNA” simulation (6. in Table 1) was used for (a), (b) and (c), while the reaction coordinate of R1 which was defined in the “r-translocation” simulation (11. in Table 1) was used for (d). (TIF) [file pone.0101951.s004.tif]

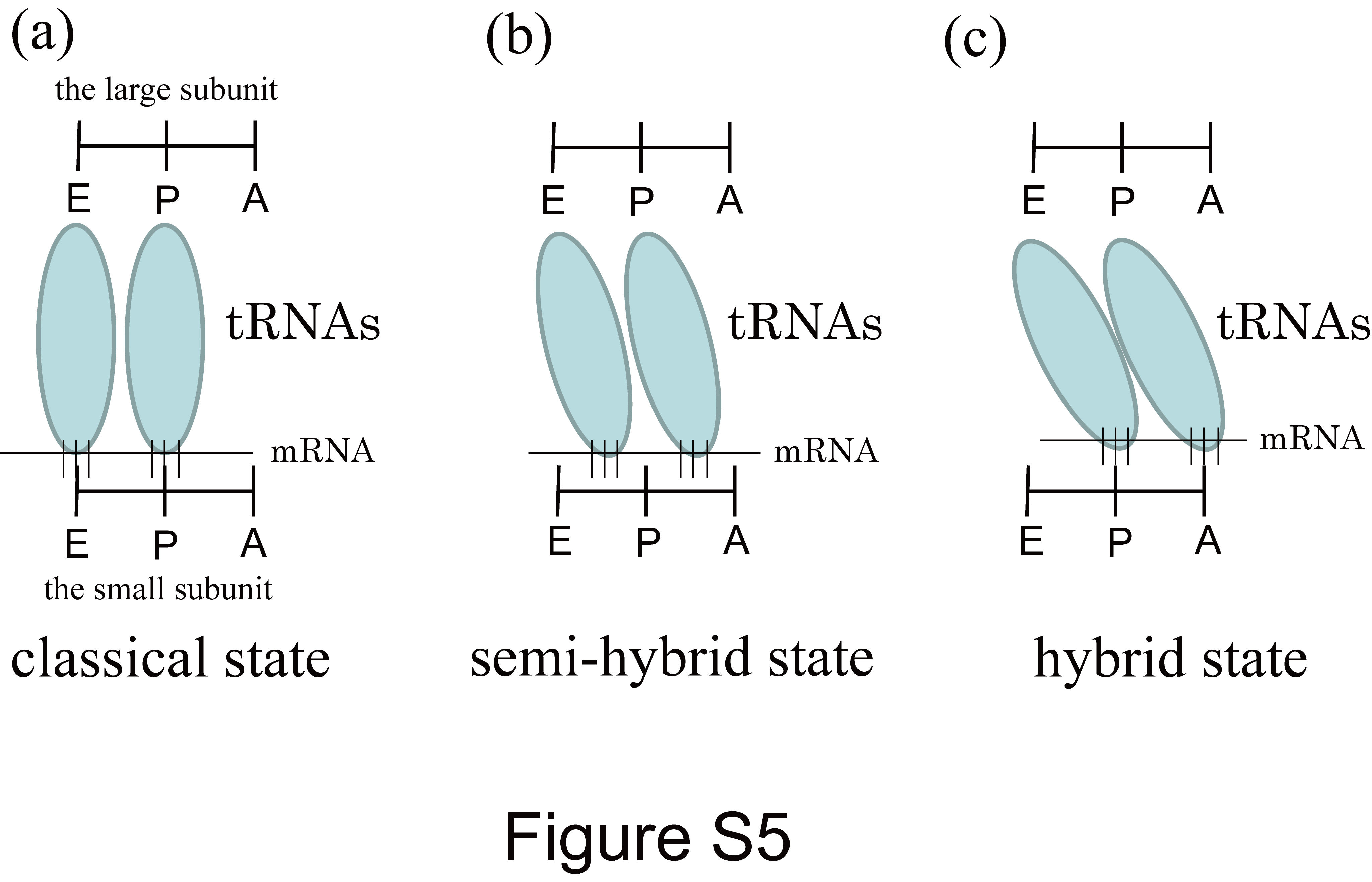

Supplement: Figure S5 — Schematic representation for the “semi-hybrid” tRNA simulation. The schematic representation for the (a) classical, (b) semi-hybrid and (c) hybrid states of tRNAs is shown. In the semi-hybrid state, the anticodons of the E- and P-tRNAs in the classical state move to between the E and P-sites and P and A-sites on the small subunit. (TIF) [file pone.0101951.s005.tif]

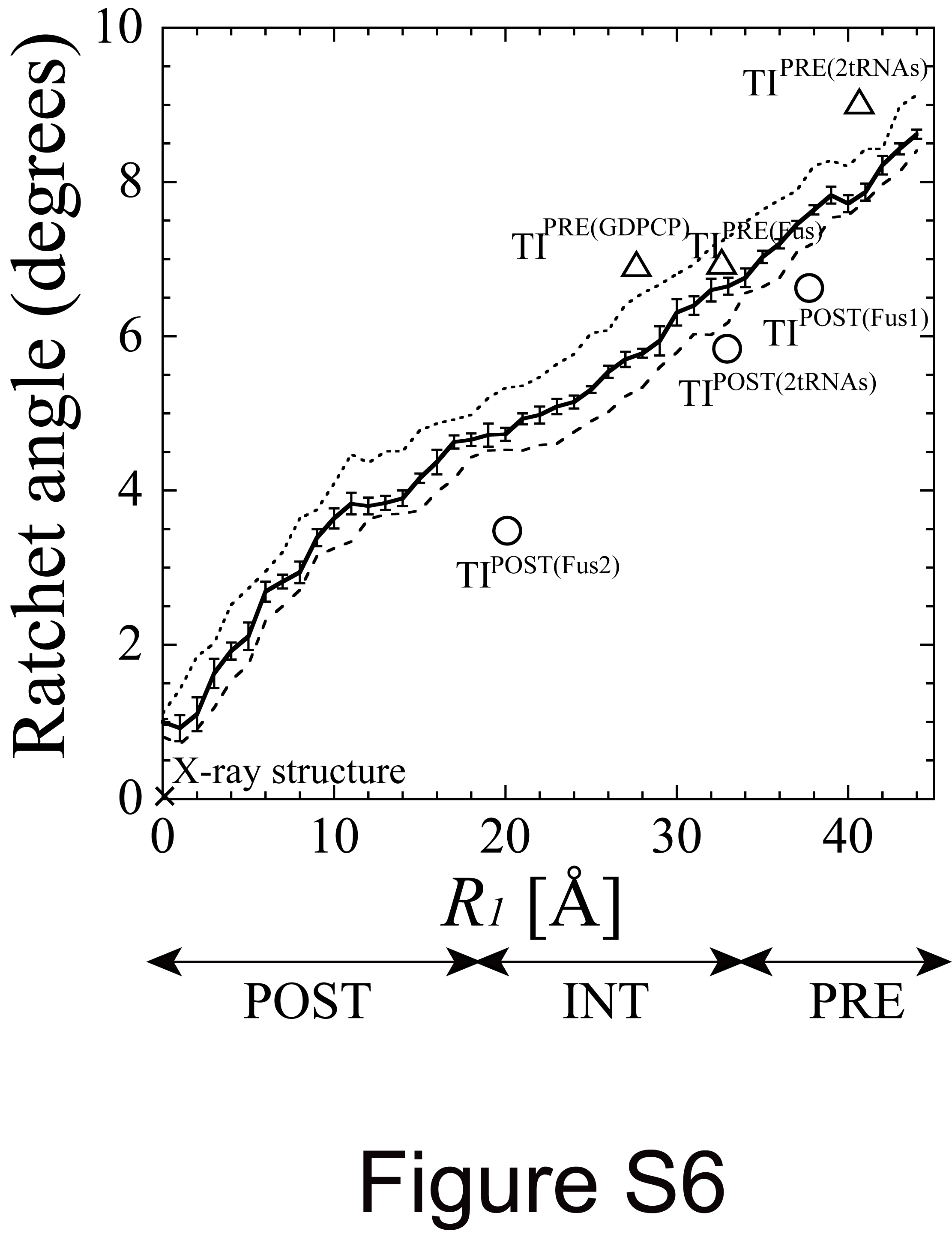

Supplement: Figure S6 — The relationship between the first reaction coordinate, R1 and the ratchet angle. The relationship between the first reaction coordinate, R1 and the ratchet angle in the first “r-translocation” simulation is shown. The maximum and minimum of the ratchet angle are shown in dotted and broken lines, respectively. For comparison, the values for TIPREs and TIPOSTs (Table 2) were plotted with triangles and circles, respectively. (TIF) [file pone.0101951.s006.tif]

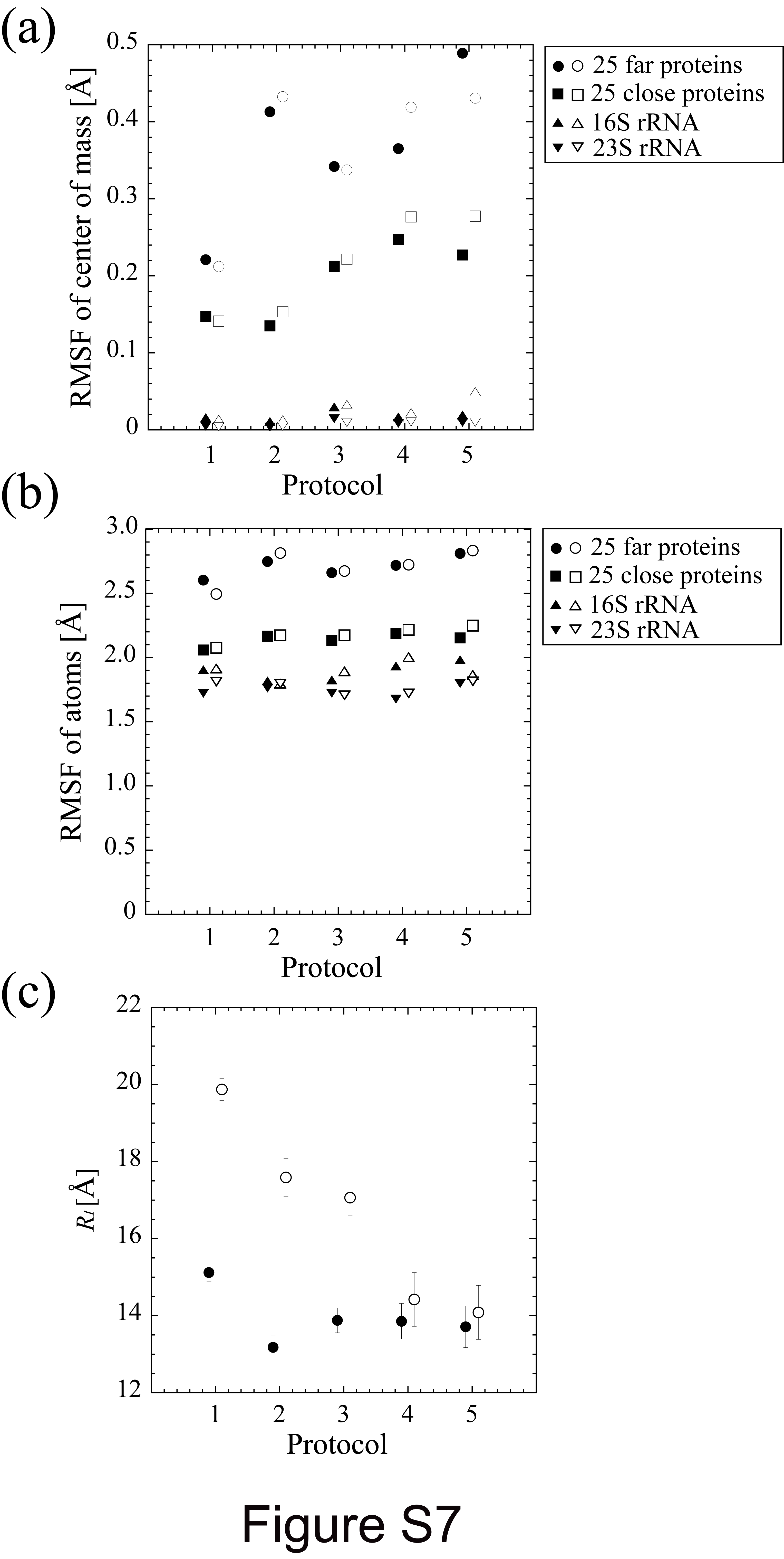

Supplement: Figure S7 — The choice of the umbrella sampling variables and the strength of their restraints. (a) The RMSFs of the centers of mass and (b) the mass-weighted RMSFs of the atoms of 16S rRNA in the small subunit, 23S rRNA in the large subunit, 25 proteins close to the center of mass of the ribosome and 25 proteins far from the center of mass of the ribosome are plotted against the protocol number. The protocol numbers 1–5 are for 50 restraints of 1.0 kcal/mol/Å2 (original), 25 restrains of 1.0 kcal/mol/Å2 for 25 close proteins, 50 restraints of 0.20 kcal/mol/Å2, 50 restrains of 0.04 kcal/mol/Å2 and no restraints on the umbrella sampling variables, respectively. The values for 16S rRNA, 23S rRNA, 25 close and far proteins are shown with triangles, inverted triangles, squares and circles, respectively. Open and closed marks are from the result of the simulations carried out at R1 = ∼15 and 21 Å, respectively. (c) The average and RMSF of R1 for protocols 1–5 are shown with open and closed circles for the simulations carried out at R1 = ∼15 and 21 Å, respectively. (TIF) [file pone.0101951.s007.tif]
