# Supplementary material for: Free-Energy Landscape of Reverse tRNA Translocation through the Ribosome Analyzed by Electron Microscopy Density Maps and Molecular Dynamics Simulations
Source: PLoS One. 2014 Jul 7;9(7):e101951. doi: 10.1371/journal.pone.0101951 (PMC4084982; doi:10.1371/journal.pone.0101951)
Supplement: Text S1 — The free-energy landscape in the classical state of tRNA (“classical tRNA” simulation). (PDF) [file pone.0101951.s008.pdf]

Text S1. The free-energy landscape in the classical state of tRNA ("classical tRNA" simulation)

Fig. S1 shows the free-energy obtained by fitting the X-ray structure (POST state) into EMD 1365 (INT state). The free energy at R_1 = 0 Å was ~ 8 kcal/mol and decreased to 0 kcal/mol at R_1 = 14 Å, indicating that the crystal structure at R_1 = 0 Å is not stable thermodynamically. In the crystal structure, the L10-L12 stalk is attached to the ribosome tightly [1]. This tight packing of the ribosome in the unit cell would make the ribosome inflexible and entropically unfavorable. From R_1 = 14 to 28 Å, the free-energy increased and went up to more than 30 kcal/mol. This indicates that the structures at R_1 > 25 Å were very unstable thermodynamically and that they are not appropriate as structures for the INT state.

Figs. S2(a-d) show the time evolution of the CC, the number of residues forming helices (α- and 3_{10}- helices) and β-sheets in ribosomal proteins and EF-G, and the Ramachandran outlier indicator during the umbrella sampling simulations. Fig. S2(b) shows that the secondary structures were maintained at the 29-th to 42-nd windows (where the average positions of R_1 in these windows, $\langle R_1 \rangle_{W=29\text{-}42}$, were more than 25.0 Å (Fig. S2(d)), corresponding to R_1 > ~ 25 Å (Fig. S1(a)). Figs S1 and S2 indicate that even though these three-dimensional structures were fit into EM density

maps and the secondary structures were maintained, they can be unstable thermodynamically. This may have occurred because of a possible over-fit of X-ray structures into lower-resolution cryo-EM density maps.

To avoid sampling the high free-energy structures in the INT state, and to search for a lower free-energy path in the INT state, we switched the target EM map from EMD-1365 (INT state) to EMD-1363 (PRE state) at $R_1 < 25$ Å. Figs. S1(b-d) show the free-energy landscape for three different cases where the target EM map was switched in the EM-fitting simulation at the 15-th, 21-st and 26-th windows (where $\langle R_1 \rangle_{W=14}$, $\langle R_1 \rangle_{W=20}$, and $\langle R_1 \rangle_{W=25}$ in the previous windows were 14.0 Å, 18.3 Å, 21.9 Å (Fig. S2(d)), and correspond to $R_1 = \sim 14$, 18 and 22 Å, respectively. These three simulations were continued until R_1 reached at $R_1 = \sim$ 27-28 Å. Among the three cases, the case at the 21-st window gave the lowest free-energy landscape at R_1 = 20-25 Å. This simulation was continued until the free-energy increased to 30 kcal/mol as shown in Fig. S1(e).

Fig. S3(a) shows the movement of the two tRNAs and EF-G in the free-energy landscape of Fig. S1(e). This figure shows that as the ribosome goes from the POST state to INT and PRE states, the anticodons of the P-tRNA, E-tRNA and domain IV of EF-G simply followed the ratchet-like movement of the ribosome by ~ 4 Å without

changing their binding sites on the small subunit. Hereafter, this simulation in which tRNAs remained in their classical states is referred to as “classical tRNA” simulation. The two reasons why tRNA did not translocate could be: (1) the EM maps and (2) the EM-fitting force. For (1) the EM structures EMD-1363 and EMD-1365 contain only a single tRNA at the P/E site [2]. Consequently, the two tRNA molecules were forced to fit into the single tRNA image, which might have caused steric stress between them and stopped their movements. For (2) to maintain the secondary structures of the ribosome, the EM-fitting force on each atom was adjusted to be on average less than 0.02 % of the atomic forces imposed by the standard all-atom energy function U_{str} in Eq. (1). This rather weak EM-fitting force was large enough to cause the ratchet-like movement because the water molecules surrounding the ribosome did not block any global motions. In contrast, the tRNAs and EF-G were surrounded by many atoms of proteins and ribosomal rRNAs. In a limited simulation time, even if there were clear images of the two tRNAs in the EM density map, this EM-fitting force would not be able to move these molecules.

Hereafter, the EM-fitting force on the tRNAs was set at zero throughout this study.

1. Gao, Y.-G., et al., *The structure of the Ribosome with Elongation Factor G Trapped*

in the Posttranslocational State. Science, 2009. **326**: p. 694-699.

2. Valle, M., et al., *Locking and unlocking of ribosomal motions.* Cell, 2003. **114**: p. 123-134.
