# Supplementary material for: Free-Energy Landscape of Reverse tRNA Translocation through the Ribosome Analyzed by Electron Microscopy Density Maps and Molecular Dynamics Simulations
Source: PLoS One. 2014 Jul 7;9(7):e101951. doi: 10.1371/journal.pone.0101951 (PMC4084982; doi:10.1371/journal.pone.0101951)
Supplement: Text S2 — Modeling of the semi-hybrid and hybrid states of tRNAs. (PDF) [file pone.0101951.s009.pdf]

Text S2. Modeling of the semi-hybrid and hybrid states of tRNAs

In the “classical tRNA” simulation, the r-translocation did not occur. Under the condition that r-translocation should occur in a limited simulation time, it would be reasonable that the anticodons of the E-tRNA and P-tRNA in the classical state are assisted to move to the P- and A-sites on the small subunits respectively during the EM-fitting simulation. The problem is that it is not known how far tRNAs move at each stage of the ratchet-like movement. One way to find out is to sample all the range of two variables of R_1 and R_2. However, this is impossible due to the heavy burden of computation. Instead, we restricted the sampling area of R_1 and R_2. For this purpose, we modeled the structures for the semi-hybrid and hybrid states, and found the coordinates of R_1 and R_2 at the lowest free-energy for these modeled structures. Then we assumed that the path for r-translocation passes through the coordinates of R_1 and R_2 at the lowest free-energies for the classical, semi-hybrid and hybrid states. In this study, to avoid confusion, the “semi-hybrid” state is used to refer to the state of tRNA between the hybrid and ~~new~~ classical states; the “semi-hybrid” state is a model state where the ancicodons of E-tRNA and P-tRNA lie between the P and E sites and between the A and P sites, respectively while they maintain the interaction with the large subunit (see the schematic representation of the semi-hybrid state in Fig. S5). It should be

noted that the concept of the “classical”, “semi-hybrid” and “hybrid” states and the concept of the “POST”, “INT” and “PRE” states are different. The former refers to the state of tRNA in the ribosome, while the latter refers to the state of the ribosome (In this study, the POST, INT and PRE states correspond to the states at R_1 = ~ 0-18 Å, ~ 19-33 Å and ~ 34-44 Å, respectively).

The positions of the anticodons of E-tRNA and P-tRNA in (1) the hybrid and PRE states, and (2) the semi-hybrid and INT states were assumed as follows. For (1) the anticodon of E-tRNA is located at the position of the anticodon of the P-tRNA in the PRE state in the “classical tRNA” simulation. The anticodon of P-tRNA is adjacent to the codon at the A-site in the PRE state in the “classical tRNA” simulation (see Fig. S5(c)). For (2) the anticodons of E-tRNA and P-tRNA are located at the middle positions between in the classical and POST states (of X-ray structure) and in the hybrid and PRE states (see Fig. S5(b)).

Steered MD (SMD) simulations were used to model the structures for the semi-hybrid and hybrid states. A SMD simulation was performed for 5 ns during which positional harmonic restraints for the anticodons were applied to the desired positions with a gradual force constant which starts from 0 kcal/mol/Å^2 and finishes at 5 kcal/mol/Å^2. To prevent the collapse of the structure of tRNAs and domain IV of

EF-G due to the possible clashes between them during the SMD simulation, a restraint to maintain the root mean square displacement (RMSD) for each of the tRNAs and domain IV of EF-G within 1 Å from their starting structures was applied. Moreover, flat-bottomed artificial distance restraints for hydrogen bonds between the anticodons of tRNAs and the corresponding codons of mRNA were additionally applied. Only when hydrogen bond distance deviated more than 0.1 Å from its ideal value, did the flat-bottomed artificial distance restraint started to work to bring it back. After 5 ns in the SMD simulation, equilibration simulation was performed for 5 ns in which the restraints for the positional restraint on the E-tRNA, P- tRNA and domain IV of EF-G were gradually removed. The EM-fitting force to maintain the structure of the ribosome at R_1 = 15 Å was also applied during the SMD simulation and the equilibration simulation.
