# Supplementary material for: Free-Energy Landscape of Reverse tRNA Translocation through the Ribosome Analyzed by Electron Microscopy Density Maps and Molecular Dynamics Simulations
Source: PLoS One. 2014 Jul 7;9(7):e101951. doi: 10.1371/journal.pone.0101951 (PMC4084982; doi:10.1371/journal.pone.0101951)
Supplement: Text S3 — The free-energy landscape in the semi-hybrid and hybrid states of tRNA (“semi-hybrid tRNA” and “hybrid tRNA” simulations). (PDF) [file pone.0101951.s010.pdf]

Text S3. The free-energy landscape in the semi-hybrid and hybrid states of tRNA (“semi-hybrid tRNA” and “hybrid tRNA” simulations)

Fig. S4(a) shows the two dimensional free-energy landscape of the P-tRNA in the “classical tRNA” simulation. To construct the atomic structure in the semi-hybrid state (Fig. S5(b)), we used the free-energy minimum structure at R_1 = 15 Å (shown as “×” for the P-tRNA in Fig. S4(a)) in the “classical tRNA” simulation. Using a SMD simulation (see Text S2), the anticodons of E-tRNA and P-tRNA were moved from the free-energy minimum to the desired positions in the semi-hybrid state (shown as “▲” for the P-tRNA in Fig. S4(b)) along the dotted arrow. Then starting from the obtained semi-hybrid tRNA structure, the same procedure as in the previous “classical tRNA” simulation was performed to obtain the free-energy landscape for the semi-hybrid state as shown in Fig. S4(b). The free-energy minimum for the semi-hybrid state was found at (R_2, R_1) = (-4.0 Å, 28 Å) (shown as “△” in Fig. S4(b)). It should be noted that the difference in the free-energy landscapes in different states is unknown because the sampled trajectories did not over-lap. Then, to obtain the optimal structure in the hybrid state (Fig. S5(c)), the anticodons of tRNAs in the semi-hybrid state at R_1 = 28 Å were moved to the desired positions in the hybrid state (shown as “●” for the P-tRNA in Fig. S4(c)). The free-energy landscape for the hybrid state was evaluated using the

same procedure as in the classical and semi-hybrid states. Fig. S4(c) shows that the free-energy minimum for the PRE state was at (R_2, R_1) = (-11.0 Å, 39 Å) (shown as "○" for the P-tRNA).
