# Supplementary material for: Free-Energy Landscape of Reverse tRNA Translocation through the Ribosome Analyzed by Electron Microscopy Density Maps and Molecular Dynamics Simulations
Source: PLoS One. 2014 Jul 7;9(7):e101951. doi: 10.1371/journal.pone.0101951 (PMC4084982; doi:10.1371/journal.pone.0101951)
Supplement: Text S4 — The verification of the “r-translocation” simulation. (PDF) [file pone.0101951.s011.pdf]

Text S4. The verification of the "r-translocation" simulation

In the "r-translocation", the movement of the anticodons of tRNAs was induced to pass through the free-energy minima in the classical, semi-hybrid and hybrid tRNA states during the EM-fitting simulation using the path-search algorithm [1]. Figs. S3(a) and S4(d) show that the path-search algorithm successfully induced the movement of the P-tRNA from the E-site and P-sites on the small subunit. Fig. S3(a) shows that at $R_1 > 37$ Å, the positions of the anticodon of the P-tRNA in the PRE state in the "classical tRNA" simulation and the anticodon of the E-tRNA in the PRE state in the "r-translocation" simulation were close to each other (also see Fig. S3(b)). This indicates the movement of the tRNAs with respect to the small subunit from the classical P/P and E/E states into the hybrid A/P and P/E states was properly directed.

1. Ishida, H., *Branch Migration of Holliday Junction in RuvA Tetramer Complex Studied by Umbrella Sampling Simulation Using a Path-Search Algorithm.* J. Comput. Chem., 2010. **31**: p. 2317-2329.
