# Supplementary material for: Free-Energy Landscape of Reverse tRNA Translocation through the Ribosome Analyzed by Electron Microscopy Density Maps and Molecular Dynamics Simulations
Source: PLoS One. 2014 Jul 7;9(7):e101951. doi: 10.1371/journal.pone.0101951 (PMC4084982; doi:10.1371/journal.pone.0101951)
Supplement: Text S5 — The choice of the umbrella sampling variables and the strength of their restraints. (PDF) [file pone.0101951.s012.pdf]

Text S5. Choice of the umbrella sampling variables and the strength of their restraints

To evaluate the choice of the restraints for the umbrella sampling variables, MD simulations using five different conditions of the restraints from the configurations at R = 15 and 20 Å were carried out: (protocol 1) 50 restraints of 1.0 kcal/mol/Å^2 (original), (protocol 2) 25 restraints for 25 proteins close to the center of mass of the ribosome-tRNAs-EFG complex (excluding L12). (The 25 proteins were selected to be S6, S11, S12, S15, S17, S18, L27, L28, L30, L32, L33, L34, L35, L36, L2, L3, L13, L14, L15, L16, L17, L19, L20, L22, L25. The distance between the centers of mass of a protein and complex was less than 89.5 Å.), (protocol 3) 50 restraints of 0.20 kcal/mol/Å^2, (protocol 4) 50 restraints of 0.04 kcal/mol/Å^2, and (protocol 5) no restraints.

For each restraint condition, two MD simulations were carried out at R_1 = ~ 15 Å (at free-energy minimum) and R= ~ 21 Å (at high free-energy barrier). The reference coordinates to fix the simulated coordinates at R_1 = 14.7 and 20.7 Å were taken from the configurations obtained in the EM-fitting simulations. The initial coordinates were taken from the configurations obtained in the sampling phase of the corresponding umbrella sampling simulations at 0 ns. For protocols 1, 2-ns trajectory in the umbrella

sampling simulation was analyzed. For protocols 2 and 3, MD simulations were carried out for 3 ns and the last 2-ns trajectories were analyzed. For protocols 4 and 5, MD simulations were carried out for 5 ns to further relax the structure of ribosome and the trajectories of the last 2-ns trajectories were analyzed.

Fig. S7(a) shows that the centers of mass of the proteins are strongly restrained to be ~ 0.1-0.2 Å for protocol 1 (original) compared with that for protocol 5 (free ribosome), ~ 0.2-0.5 Å. (It should be noted that there is no restraint for 16S rRNA and 23S rRNA.) In contrast, Fig.S7(b) shows that the mass-weighted RMSFs of the atoms of the proteins were for original (protocol 1) were ~ 2.0-2.6 Å, which were not very different from those in the free ribosome (protocol 5), ~ 2.4-2.8Å. However, as the RMSFs of the atoms in original (protocol 1) decreased by ~ 10% compared with those in the free ribosome (protocol 5), the efficiency of the conformational sampling was thought to have deteriorated. In terms of the sampling efficiency, the choice of 25 restraints (protocol 2) could have been better than the original choice of 50 restraints (protocol 1). Although further less restraints (such as in protocols 3 and 4) showed larger RMSF of atoms (i.e. better conformational sampling), Fig. S7(c) shows that R_1 significantly decreased from the desired position to be fix at R_1 =20.7 Å for the simulation at R_1 = ~ 21 Å. This indicates that the structure went down from the high

free-energy barrier at R_1 = ~ 21-22 Å. Therefore, if weaker restraints were used then more repetition of the EM-fitting and umbrella sampling simulations would be required for the structure to overcome the free-energy barrier at R_1 = ~ 21-22 Å. Too weak restraints in protocol 4 would fail to enable the structure to overcome the free-energy barrier.
