# Supplementary material for: Free-Energy Landscape of Reverse tRNA Translocation through the Ribosome Analyzed by Electron Microscopy Density Maps and Molecular Dynamics Simulations
Source: PLoS One. 2014 Jul 7;9(7):e101951. doi: 10.1371/journal.pone.0101951 (PMC4084982; doi:10.1371/journal.pone.0101951)
Supplement: Text S6 — The effect of different ion force-fields on the free-energy landscape. (PDF) [file pone.0101951.s013.pdf]

Text S6. The effect of different ion force-fields on the free-energy landscape

In this study, the AMBER parm99SB force-field for the monovalent K^+ and Cl^- ions was used although the force-field parameters for these ions can cause artifacts in MD simulations due to the strong ion-ion interactions [1] [2]. To estimate the influence of the force-filed for K^+ and Cl^- ions on the free-energy landscape, another MD simulation was carried out using a corrected force-field which was developed by Joung and Cheatham [3]. The MD simulation was started from the same structure used for the second "r-translocation" simulation. The ions and water molecules were located around the ribosome and were equilibrated using the same procedure as the previous system (see Method) except that the positional restraints on all the heavy atoms in the ribosome-tRNA-EFG complex were imposed during the equilibration. (The system-size was set to be the same as the previous system during the NTP simulation).

The umbrella sampling simulation for the new system was carried out from R_1 = ~ 20 Å (Table I) as follows: (1) instead of using the EM-fitting simulation, a steered molecular dynamics (SMD) simulation was carried out during which positional restraints on all the heavy atoms in the ribosome-tRNAs-EFG complex were imposed from 0 to 0.5 kcal/mol/Å^2 in 500 ps to match the structure to that which was obtained by the EM-fitting simulation for the previous system. Then the umbrella sampling

simulation was carried out using exactly the same procedure as the previous one (see Method). Fig. S2(h) shows that the free-energy from this system was not significantly different from the previous result of Fig. S2(g) (or Fig. 2). The average of absolute difference between each value of their free-energies in the range of R_1 = 20-41 Å was 1.3 kcal/mol, while the average of absolute difference between first and second "r-translocation" simulations was 2.2 kcal/mol. This means that the effect of different ion force-fields on the free-energy landscape along the same trajectory was smaller than the effect of different trajectories on the free-energy landscape. Therefore, it is thought that the effect of the different ion force-fields on the free-energy landscape was not significant in this study.

1. Vaiana, A.C., E. Westhof, and P. Auffinger, *A molecular dynsmics simulation study of an aminoglycoside/A-site RNA complex.* Biochimie, 2006. **88**: p. 1061-1073.

2. Auffinger, P., T.E. Cheatham III, and A.C. Vaiana, *Spontaneous formation of KCl aggregates in biomolecular simulations: a force field issue?* J. Chem. Theory Comput., 2007. **3**: p. 1851-1859.

3. Joung, I.S. and T.E. Cheatham, *Determination of alkali and halide monovalent ion parameters for use in explicitly solvated biomolecular simulations.* J. Phys. Chem. B, 2008. **112**: p. 9020-9041.
