# Supplementary material for: Free-Energy Landscape of Reverse tRNA Translocation through the Ribosome Analyzed by Electron Microscopy Density Maps and Molecular Dynamics Simulations
Source: PLoS One. 2014 Jul 7;9(7):e101951. doi: 10.1371/journal.pone.0101951 (PMC4084982; doi:10.1371/journal.pone.0101951)
Supplement: Text S7 — Available atomic models of the translocational ribosome obtained using molecular dynamics simulations and cryo-EM density maps. (DOCX) [file pone.0101951.s014.docx]

**Text S7, “Available atomic models of the translocational ribosome obtained using molecular dynamics simulations and cryo-EM density maps”**

First, (1) an initial structure at *R_1_* = 0 Å, (2) a structure fitted into EMD-1365 *R_1_* = ~ 30 Å (CC in Eq. (2) = 0.847) in the “classical-tRNA” simulation, (3) a transitional structure at *R_1_* = ~ 26 Å and (4) a structure fitted into EMD-1363 at *R_1_* = ~ 43 Å (CC = 0.843) in the “r-translocation” simulation were selected. Second, energy minimization was carried out to alleviate unfavorable interactions in the system which may have been caused by the EM-fitting and umbrella sampling simulations. The protocols for the energy minimization and molecular dynamics simulation are the same as that used in MATERIALS AND METHODS “Molecular dynamics simulation with explicit water molecules” unless they are specifically mentioned otherwise. Third, The system was heated from 0 K to 300 K within 500 ps, during which the water molecules and ions were allowed to move freely. The system was equilibrated for 1.5 ns with decreasing restraints on the molecules for 1 ns, and for 500 ps with no restraint at a constant temperature of 300 K. Finally, the relaxed structures (1-4) were best-fitted to the energy-minimized structures which were obtained at the second stage in order to remove the artificial movements of the translational and rotational movements of the ribosome-tRNAs-mRNA-EFG complexes. The CCs for the relaxed structures of the ribosome-tRNAs-mRNA-EFG complex were 0.845 and 0.840 for (2) and (4), respectively.

In addition to these structures (1-4), structures (2) and (4) were fitted into the actual EMD-1365 and EMD-1363 respectively. (Use Chimera software [[1](#_ENREF_1)] to see their match.) The procedure for the transformation of structures (2) and (4) to the actual EM-density maps is the reverse of transformation from the EM density maps to the initial structure at *R_1_* = 0 Å. (Transformation from the actual EM density maps to the initial structure at *R_1_* = 0 Å is given in MATERIALS AND METHODS “Alignment of the EM density maps to the initial atomic model”.)

It should be noted that the movements of the E-tRNA, P-tRNA and EF-G in structure (2) are not by tRNA translocation but by the ratchet-like movement of the ribosome.

Comparing structure (4) and the atomic model in the pre-translocational state (PDB code: 3J5X/3J5W) constructed from EMD-5800 [[2](#_ENREF_2)], the location of the P-tRNAs is different from each other. The T-arm of the P-tRNA in 3J5X/3J5W is located to interact rather with H38 not H84 in the large subunit, while the T-arm of the P-tRNA in structure (4) is located to interact rather with H84 not H38. The state of the former is likely to correspond to pre4 of the pre-transitional state (from pre1 to pre5) observed in the time-resolved EM experiment of reverse translocation [[3](#_ENREF_3)]. The state of the latter is likely to correspond to pre5, which connects to post1 of the post-transitional state (from post1 to post3).

1. Pettersen, E.F., et al., *UCSF Chimera—a visualization system for exploratory research and analysis.* J. Comput. Chem., 2004. **25**: p. 1605.

2. Brilot, A.F., et al., *Structure of the ribosome with elongation factor G trapped in the pretranslocation state.* Proc. Natl. Acad. Sci. U.S.A., 2013. **110**: p. 20994-20999.

3. Fischer, N., et al., *Ribosome dynamics and tRNA movement by time-resolved electron cryomicroscopy.* Nature, 2010. **466**: p. 329-333.
